# Ultra-Efficient Saline Soil Remediation with Passive Multistage Solar Distiller via Water Recycling

**DOI:** 10.1007/s40820-026-02207-7

**Published:** 2026-05-08

**Authors:** Junhui Li, Jiebin Tang, Guangchun Yang, Dengchao Liang, Yi Wang, Wenwen Zhao, Xin Xia, Weijia Zhou, Yafang Zhang, Dongjin Xin, Guobin Xue

**Affiliations:** 1 Institute for Advanced Interdisciplinary Research (iAIR), School of Chemistry and Chemical Engineering, University of Jinan, Jinan, 250022 People’s Republic of China; 2 School of Physics and Technology, University of Jinan, Jinan, 250022 People’s Republic of China; 3 Shandong Key Laboratory of Ubiquitous Intelligent Computing, School of Information Science and Engineering, University of Jinan, Jinan, 250022 People’s Republic of China

**Keywords:** Saline soil remediation, Passive multistage solar distiller, Latent heat recovery, Water recycling, Edge salt rejection

## Abstract

The remediation of saline land is of great strategic significance for ensuring food security and promoting ecological restoration. Interfacial solar evaporator shows considerable potential in soil remediation but still faces challenges, such as low solar-steam efficiency and high freshwater consumption. Herein, we report for the first time a passive multistage solar distiller for ultra-efficient saline soil desalination that integrates the latent heat recovery, water recycling and edge salt rejection. Under one-sun irradiation (1 kW m^−2^), the 7-stage solar distiller achieves a water yield of 3.0 kg m^−2^ h^−1^ with a solar-steam efficiency of 200% in saline soil. After 12 days of treatment, the salinity in 30 cm-thick saline soil had decreased from 22.5 to 2.33 g kg^−1^, with a water consumption (water-to-soil ratio) of 40.1 kg m^−2^. Furthermore, the large-scale saline soil desalination using the multistage solar distiller works efficiently outdoors, indicating that such technology can promote the green remediation of global saline land and the sustainable development of agriculture.

**Supplementary Information:**

The online version contains supplementary material available at 10.1007/s40820-026-02207-7.

## Highlights

- The passive multistage solar distiller can ultra-efficiently desalinate saline soil.
- The strategy features latent heat recovery, water recycling, and edge salt rejection.
- We desalinated 30 cm-thick saline soil from 22.5 to 2.33 g kg^-1^ within 12 days, with a water consumption (water-to-soil ratio) of 40.1 kg m^-2^.

**Supplementary Information:**

The online version contains supplementary material available at 10.1007/s40820-026-02207-7.

## Graphical abstract

**Figure:**
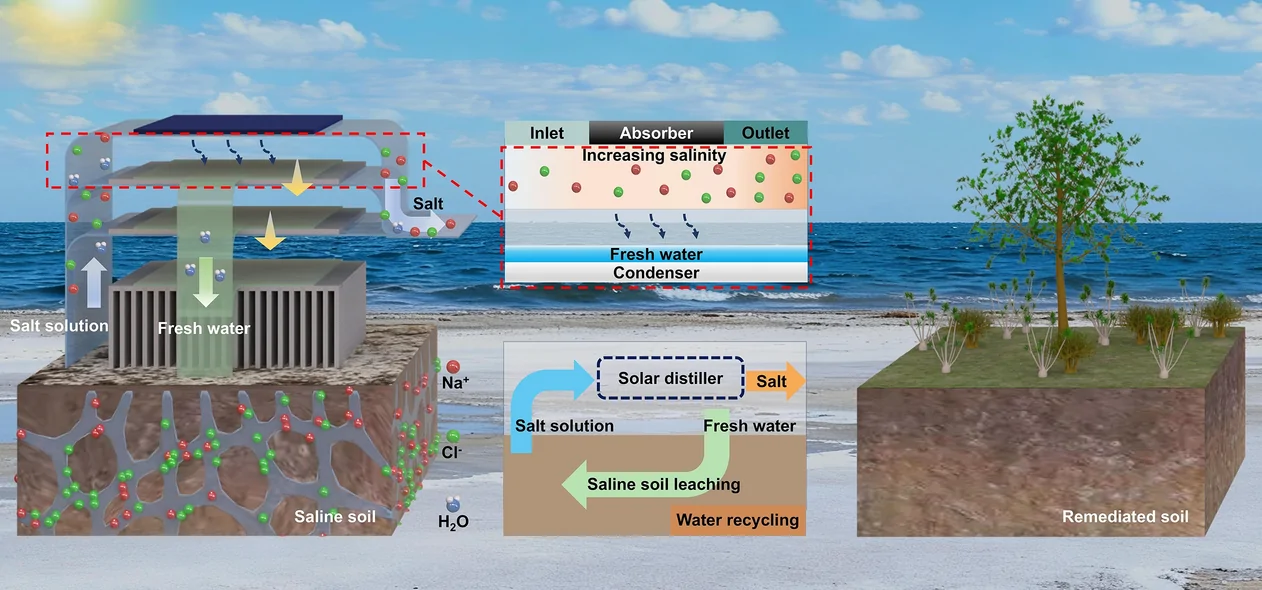

## Introduction

Soil salinization poses a global ecological challenge, affecting approximately 1 billion hectares of land worldwide [1–4]. Coastal saline soils are mainly distributed in the eastern coastal regions of China, covering a total area of approximately 3.27 million hectares. As one of the major saline soil types in China, they serve as an extremely precious national resource of potential reserved arable land. Coastal saline soil severely impairs plant growth by creating a high osmotic environment that disrupts water uptake in plants, thereby ultimately leading to reduced growth and lower crop yields [5–8]. Thus, researchers have developed multiple strategies for saline soil remediation [9–15]. Irrigation leaching is the most widely adopted method [16–22], in which soil salinity is reduced by leaching substantial amounts of freshwater to leach soluble salts downward into deeper soil strata from the root zone or drain them out of the fields. Irrigation leaching demands substantial amounts of freshwater, while coastal regions are confronted with freshwater resources shortages due to intense surface evaporation and seawater intrusion, thereby exacerbating pressures on the water-land nexus. Crucially, saline effluent discharge poses a direct threat to adjacent marine and estuarine ecosystems.

In recent years, the interfacial solar evaporator has shown considerable potential in soil remediation [23–31]. The interfacial evaporation process ensures the high solar-steam efficiency since the thermal energy converted from sunlight with the photothermal material is localized at the evaporation interface [32–37]. When an interfacial solar evaporator is used in saline soil remediation, saline water present in the soil is transported via capillary action to the evaporation interface by a water-absorbing material. With the water evaporation, the salt from the saline soil is enriched within the evaporator and thus reduces the salinity in the soil. Compared to traditional irrigation-leaching methods, interfacial solar evaporation technology enables in-situ, efficient remediation of saline land while significantly reducing freshwater consumption. Wu et al. successfully fabricated a biomimetic leaf-like three-dimensional polypyrrole-coated photothermal evaporator (NW-PPy) [38], which stably operates for 10 consecutive days outdoors and reduces the overall soil salinity by approximately 80%. Our research group previously developed a biomimetic solar-driven salt extractor with carbon nanotube-sodium polyacrylate hydrogel (CNT-PAAS) [39] as a salt-resistant evaporation layer. Under real outdoor conditions, the device operated stably for 7 consecutive days, reducing soil salinity from 27 to 1.4 g kg^−1^, achieving a desalination ratio of 95.2%. However, the thermal evaporation process inherently faces the challenge of high latent heat of water vaporization, which limits the evaporation rate even under conditions of high solar-steam efficiency. Due to water serving as the primary medium for salt transport, a limited evaporation rate directly reduces the salt flux from the soil. The continuous evaporation of water into the atmosphere necessitates sustained freshwater input to maintain system equilibrium, leading to significant freshwater consumption. In addition, as an emerging technology, this strategy is still restricted to shallow soil layers and laboratory-scale applications. Nevertheless, a soil depth of approximately 30 cm is typically required to satisfy the cultivation conditions of numerous crops (e.g., wheat, rice, maize and soybeans) [40–42]. Its scalability and adaptability have not yet been quantitatively evaluated under real-field conditions, which involve spatially variable soil thickness and field-scale remediation demands.

Here, we propose to desalinate saline soil with a passive multistage solar distiller [43–50]. The latent heat of water evaporation is reused with multiple stages, enabling the device to demonstrate a significantly high solar-steam efficiency and evaporate more saline water under a given solar input. This enhanced evaporation process accelerates the extraction of salt from the saline soil, thereby improving the overall desalination rate. Simultaneously, the fresh water generated in this distiller is recycled to the saline soil, resulting in a substantial reduction in total freshwater consumption. Furthermore, the evaporator was specifically designed to localize salt crystallization to a dedicated extended zone, effectively maintaining a low salt concentration in the primary evaporation zone and enabling continuous, stable operation without salt clogging. Under one-sun irradiation, the solar distiller achieved a remarkable solar-steam efficiency of 200%, equivalent to a water production rate of 3.0 kg m^−2^ h^−1^. To ensure the saline water supply to the distiller from 30 cm-thick saline soil, a U-tube was designed to optimize the water supply pathway. The soil salinity can be reduced from 22.5 to 2.33 g kg^−1^ in 12 days. Results from large-scale outdoor experiments demonstrated that the saline soil in a 55 cm × 18 cm × 17 cm container was desalinated to 2.3 g kg^−1^ within 14 days. This technology provides a viable and systematic solution for the ecological remediation of global saline land.

## Experimental Procedures

### Materials

Commercial solar absorber was supplied by Dezhou Jinheng New Energy Co., Ltd. Nonwoven fabric and insulating foam were purchased from www.Taobao.com. The coastal saline soil was collected from Dongying City, Shandong Province, China (118.9° E, 37.6° N). Carbon nanotube (CNT) powder was provided by Chengdu Organic Chemicals Co., Ltd.

### Material Characterization

The morphological features of the samples were examined using scanning electron microscopy (SEM; Hitachi Regulus 8100). Optical absorption of the samples over the wavelength range of 300-2500 nm was measured with a Hitachi UH4150 spectrophotometer. X-ray diffraction (XRD) patterns of the saline soil were obtained using a Bruker AXS D8 Advance diffractometer.

### Preparation of CNT-Coated Fabric

CNT-coated fabric was prepared by immersing nonwoven fabric in a 10 mg mL^−1^ CNT suspension for 5 min, followed by drying in an oven at 60 °C for 30 min. To improve the uniformity and density of the CNT coating, the immersion-drying procedure was repeated twice. The resulting CNT loading was 1.5 mg cm^−2^.

### Soil Preparation

Soil sourced from saline land was dried at 60 °C and then passed through a 2 mm nylon sieve. Four soil samples with moisture contents of 10%, 20%, 30%, and 40% were prepared by mixing dried soil with deionized water to evaluate the water absorption performance of the nonwoven fabric. For the traditional irrigation experiments, dried soil was used. In all solar-driven desalination tests, including laboratory-scale thin soil, laboratory-scale thick soil, and outdoor large-scale thick soil experiments, soil with 40% water content was employed. In the traditional irrigation and laboratory-scale thin-layer soil desalination tests, 180 g of soil was placed in a 6 cm × 6 cm × 5 cm acrylic cubic container. For the laboratory-scale thick-layer desalination tests, 1080 g of soil was packed into a 6 cm × 6 cm × 30 cm acrylic container. In the outdoor large-scale experiments, 17 kg of soil was placed in a 55 cm × 18 cm × 17 cm container.

### Solar-Driven Soil Desalination Procedure

The laboratory desalination devices were positioned above the fully sealed soil containers, with the nonwoven fabric inserted to supply water. Evaporation tests were conducted under irradiation from a uniform xenon lamp light source (CEL-PF3000-TEB) equipped with an AM 1.5 filter. The light intensity at the evaporation surface was calibrated using a CEL-NP2000 optical power densitometer. Temperature was recorded continuously using thermocouples connected to a data logger (TC-08) at one-second intervals. Spatial temperature distributions were captured with an infrared camera (FLIR E64501). Mass change was measured accurately using a Mettler-Toledo analytical balance (ME204) and recorded at 5 s intervals. All indoor evaporation tests were performed under ambient laboratory conditions (approximately 25 °C and 50% relative humidity) using deionized water throughout the experiments.

### Salt Salinity Measurement of the Fabric

Prior to device assembly, the mass and dimensions of the nonwoven fabric and the CNT-coated fabric were measured. After a specific operating period, the fabric was cut into uniform segments along its length. The area of each segment was measured, after which they were weighed and then dried. The salinity of fabric ω_f_ was calculated as follows:

1 $${\upomega}_{{\mathrm{f}}} = \frac{{{\mathrm{m}}_{2} - \frac{{\mathrm{A}}}{{{\mathrm{A}}_{0} }} \cdot {\mathrm{m}}_{0} }}{{{\mathrm{m}}_{1} - \frac{{\mathrm{A}}}{{{\mathrm{A}}_{0} }} \cdot {\mathrm{m}}_{0} }} \times 100{{\% }}$$

In model-I, m_0_ denotes the mass (g) of the dry CNT nonwoven fabric, in the remaining models, m_0_ denotes the initial mass (g) of the fabric. Furthermore, m_1_ denotes the mass (g) of the wet sample after evaporation, m_2_ denotes the mass (g) of the dried sample, A denotes the sample area (cm^2^), and A_0_ denotes the initial fabric area (cm^2^).

### Measurement of the Soil Salinity

Soil salinity was determined by collecting a 1 cm^3^ soil sample from the vicinity of the nonwoven fabric and drying it in an oven. The dried soil was then mixed with deionized water at a mass ratio of 1:5. After thorough settling, a portion of the supernatant was extracted and dried. The soil salinity (ω_s_) was calculated using the following equation:

2 $${\upomega}_{{\mathrm{s}}} = \frac{{\left( {{\mathrm{m}}_{1} - {\mathrm{m}}_{0} } \right) \times {\mathrm{D}}}}{{\mathrm{m}}}$$

where m₁ is the total mass of the dried supernatant and the petri dish (g), m₀ is the mass of the petri dish (g), m is the mass of the dry soil sample (g), and D is the dilution factor (mass ratio of the total water used to the mass of the supernatant extracted).

### Solar-Steam Efficiency of the Multistage Solar Distiller

Solar-steam efficiency (η) of the multistage solar distiller was calculated using the following equation:

3 $${\upeta } = \frac{{{\dot{\mathrm{m}}}_{{{\mathrm{evap}}}} \times {\mathrm{h}}_{{{\mathrm{fg}}}} }}{{{\mathrm{q}}_{{{\mathrm{sun}}}} }} \times 100{{\% }}$$

4 $${\dot{\mathrm{m}}}_{{{\mathrm{evap}}}} = \frac{{{\mathrm{m}}_{{{\mathrm{evap}}}} }}{{\mathrm{A}}}$$

where ṁ_evap_ is the water production rate per unit area (kg m^**−**2^ h^**−**1^), h_fg_ is the enthalpy of vaporization (kJ kg^**−**1^), q_sun_ is the incident solar power density (kW m^**−**2^), m_evap_ is the water production rate (kg h^−1^), A is area of the evaporation zone (m^2^).

### Measurement of the Water Consumption

We simultaneously treated identical saline soil with a water content of 40% using three strategies (irrigation leaching, interfacial evaporation, and multistage distillation). The samples treated by interfacial evaporation and multistage distillation were, respectively, placed on electronic balances, and the mass loss of the samples was recorded in real time, which represented the water consumption during the saline soil reclamation process. To maintain a continuous and stable evaporation process, deionized water equivalent to the mass loss was added to the samples. When the salinity of all three soil samples decreased to 1.5 g kg^−1^, the total amount of supplemented water (m_intro-water_) for each group was recorded. Water consumption (M_consum-water_) for one treatment method is calculated using the following formula:

5 $${\mathrm{M}}_{{{\mathrm{comsum}} - {\mathrm{water}}}} = \frac{{{\mathrm{m}}_{{{\mathrm{intro}} - {\mathrm{water}}}} }}{{{\mathrm{A}}_{{{\mathrm{soil}}}} }}$$

where m_intro-water_ is the total amount of supplemented water introduced into the system, A_soil_ is the surface area of the soil.

## Results and Discussion

### Passive Multistage Solar Distiller for Ultra-Efficient Saline Soil Remediation

**Fig. 1:**
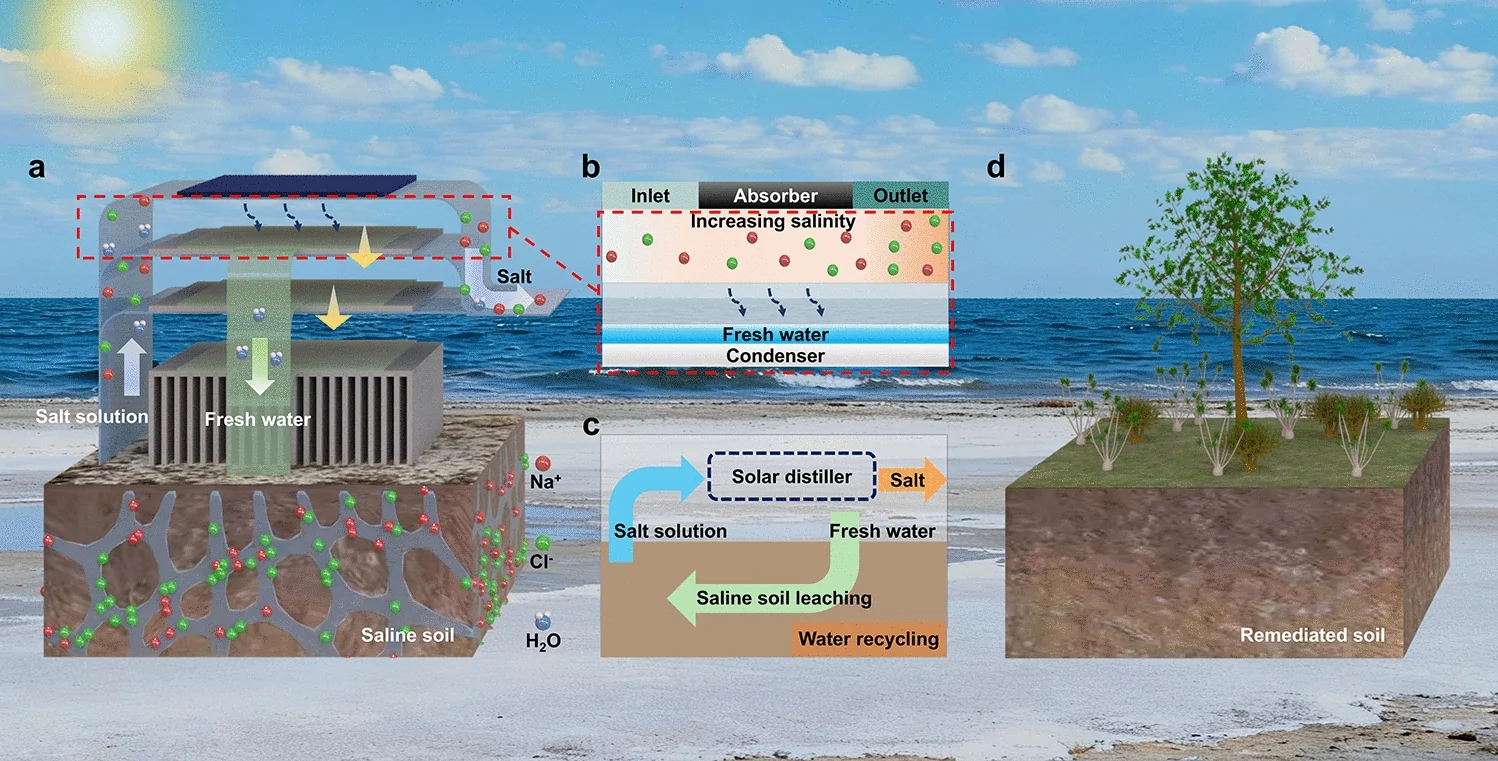
Multistage solar distiller for saline soil desalination. **a** Schematic of the solar distiller working on saline soil. The saline water from the soil was desalinated with the multistage solar distiller and the salt is enriched at the edge of the evaporator, thus reducing the salinity of the saline soil. **b** Schematic diagram of the salinity distribution within the nonwoven fabric of the evaporation layer. **c** Schematic of the water circulation loop in which the desalinated water was recycled to flush the soil. **d** Treated saline soil becomes suitable for plant cultivation

As shown in Fig. 1a, a solar-powered multistage distillation system has been applied to saline land remediation. The system consists of a photothermal absorber and a multistage distillation unit, wherein the absorber converts solar energy into thermal energy to drive the multistage distillation process below. The latent heat released during water evaporation is reused to significantly enhance the overall solar-steam efficiency. In each stage of the distiller, the upper nonwoven fabric absorbs saline water from the soil, while the lower nonwoven fabric transports the distilled freshwater out. As shown in Fig. 1b, during the transport of the saline solution through the upper non-woven fabric, the salt concentration gradually increases with water evaporation. We extend the upper nonwoven fabric beyond the distiller, allowing salt to accumulate and crystallize outside the distiller. The salt concentration within the distiller is maintained at a low level, thereby preventing salt clogging inside the distiller and ensuring the operational stability. Meanwhile, the water collected by the lower nonwoven fabric is recycled to flush the soil, significantly reducing water consumption (Fig. 1c). After several days of treatment, the salinity in saline soil decreases to levels suitable for plant cultivation (Fig. 1d).

**Fig. 2:**
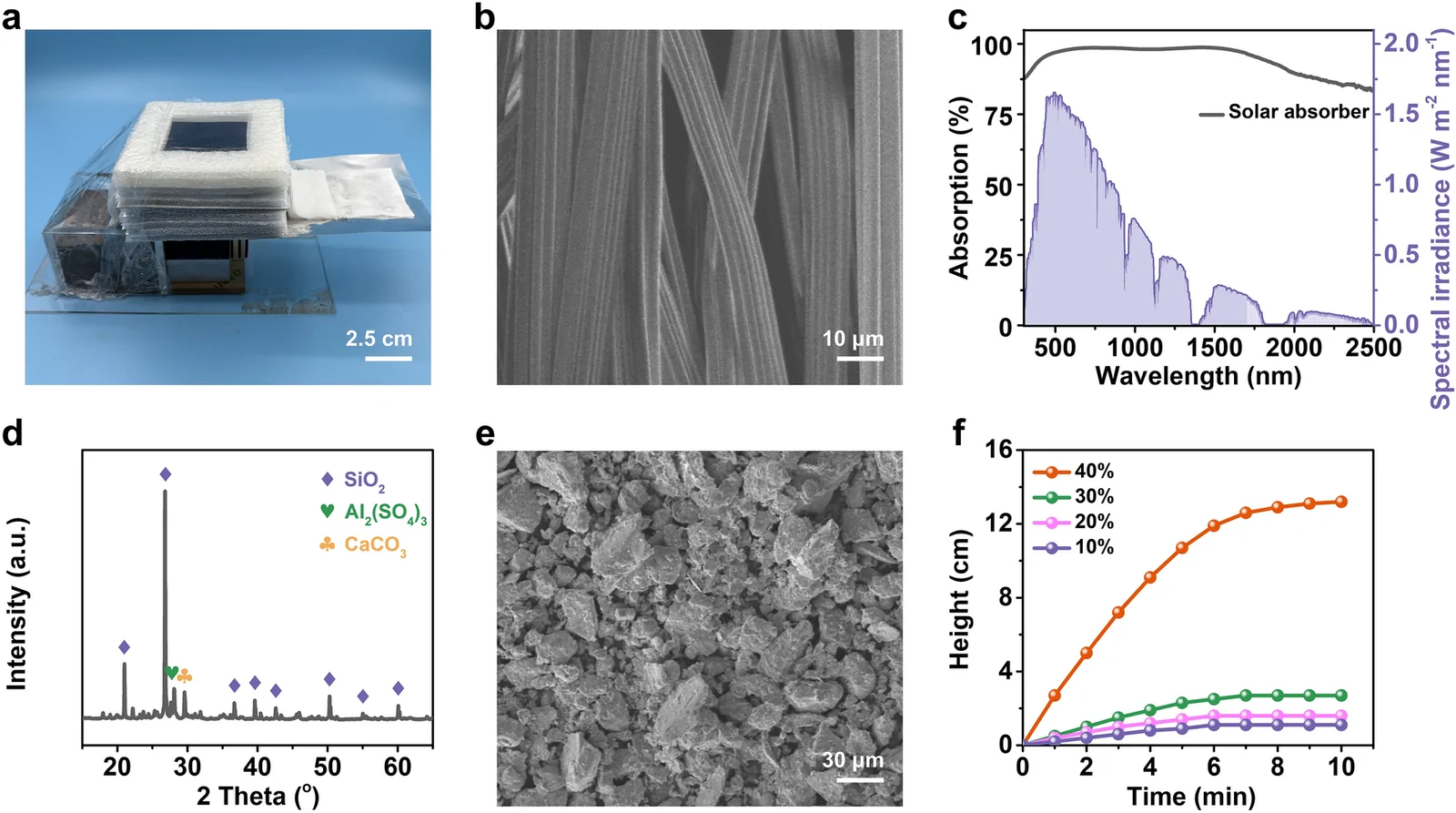
Material characterization. **a** Photograph of the multistage solar distiller for saline soil desalination. **b** SEM image of the nonwoven fabric. **c** Absorption spectrum of the absorber in the wavelength ranging from 300 to 2500nm. **d** XRD pattern of the soil. **e** SEM image of the porous soil. **f** Water absorption height of the fabric in soil with different water contents

Figure 2a shows a photograph of the multistage solar distiller for saline soil remediation. Nonwoven fabric composed of cellulose fibers with diameters of approximately 10 μm is used to absorb water from the saline soil (Fig. 2b). A commercially available solar selective absorber with a low emissivity of 5% and high solar absorption of 95% was used here (Fig. 2c). To suppress convective heat loss, the absorber is covered with transparent plastic wrap. The soil consists primarily SiO_2_, Al_2_(SO_4_)_3_, and CaCO_3_ (Fig. 2d). Figure 2e shows the SEM image of soil particles, ranging from about 10 to 100 μm in diameter. During the saline soil remediation process, a continuous capillary flow forms between the porous soil and the nonwoven fabric. The water absorption height of the fabric is directly influenced by the water content of the soil. As shown in Fig. 2f, the water absorption height of the fabric is about 13.3 cm in saturated soil (40% water content) (Fig. S1). As the soil water content decreases, the capillary channels between the fabric and soil are damaged, thus the water absorption height will be reduced. And as shown in Fig. S2, when the soil moisture content is relatively low the evaporation rate of the device decreases substantially. Therefore, to maintain a high evaporation rate, we adopted a soil moisture content of 40% in all subsequent desalination tests. Meanwhile, to maintain a high soil moisture content, condensed water from our distillation device is recycled back to the soil, and freshwater is continuously replenished to offset water loss during operation.

### Design Evolution of the Solar Evaporator for Saline Soil Remediation

**Fig. 3:**
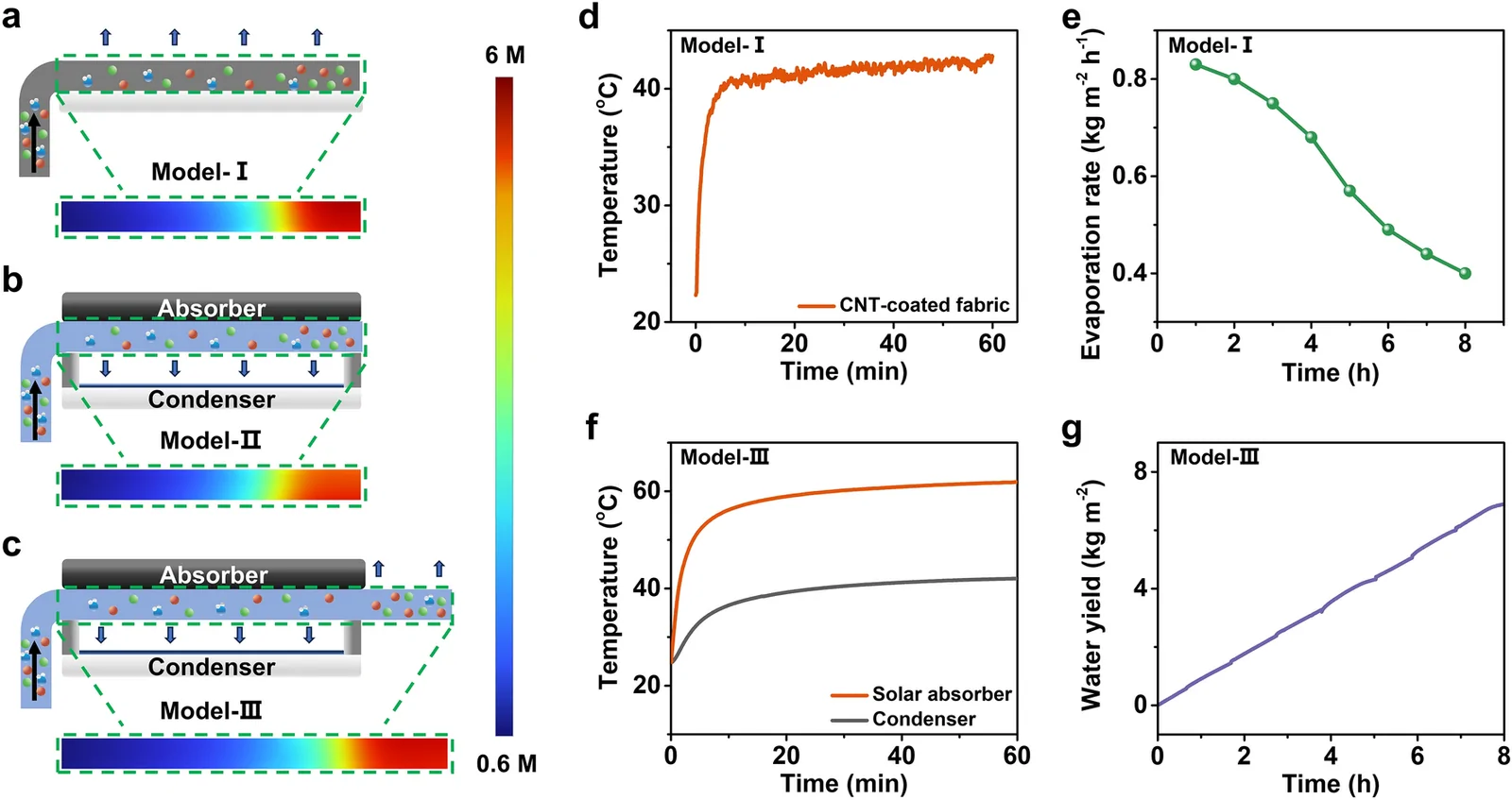
Design evolution of the solar evaporator for saline soil remediation. **a**-**c** Schematics of model-I, model-II and model-III device. The bottom is the concentration distributions in the fabric. **d** Temperature of the evaporator during soil desalination with model-I. **e** Time-dependent evaporation rate in model-I during soil desalination without additional water supply. **f** Temperature of the absorber and condenser during soil desalination in model-III. **g** Water production rate in model-III during soil desalination without additional water supply. It should be noted here that the collected water is recycled and returned to the soil per hour during this testing process

The conventional interfacial solar evaporator, designated as model-I (Note S1 and Figs. S3–S9), is shown in Fig. 3a. During the saline soil desalination process, saline water from the soil is transported to the evaporation interface and directly evaporates into the air under solar illumination. As water evaporates, salts accumulate in the surface of the evaporator, thereby enabling continuous soil desalination. The simulated salinity distribution is shown in the bottom of Fig. 3a, alongside the experimental salinity distribution (Fig. S10a, b). The model-I device can achieve an evaporation rate of 0.88 kg m^−2^ h^−1^ at approximately 40 °C (Figs. 3d and S6). Due to the continuous depletion of water in the soil, the evaporation rate gradually decreases (Fig. 3e). Consequently, to maintain stable operation in this mode, fresh water must be continuously supplemented. As shown in Fig. 3b, water vapor should be collected to reduce water consumption, thereby enabling the design of model-II (Note S2 and Figs. S11-S15). In this configuration, as water evaporates, salts accumulate in the fabric in the inner part of the distiller, causing salt clogging (Figs. 3b and S10a, c). As illustrated in Fig. 3c, to enhance operational stability, the fabric was extended outward from the distiller, resulting in the construction of model-III (Note S3 and Figs. S16–S20). In this configuration, salt accumulation occurs at the edge of the fabric, preventing clogging inside the distiller (Figs. 3c and S9a, d). The temperature of model-III is shown in Fig. 3f, where the evaporation layer reached 59 °C within 20 min, with a water production rate of 0.89 kg m^−2^ h^−1^ (Fig. S18a). We recycled this collected water into the saline soil to maintain soil moisture, ensuring a stable distillation process for eight consecutive hours (Figs. 3g and S19).

### Soil Desalination Using the Multistage Solar Distiller

**Fig. 4:**
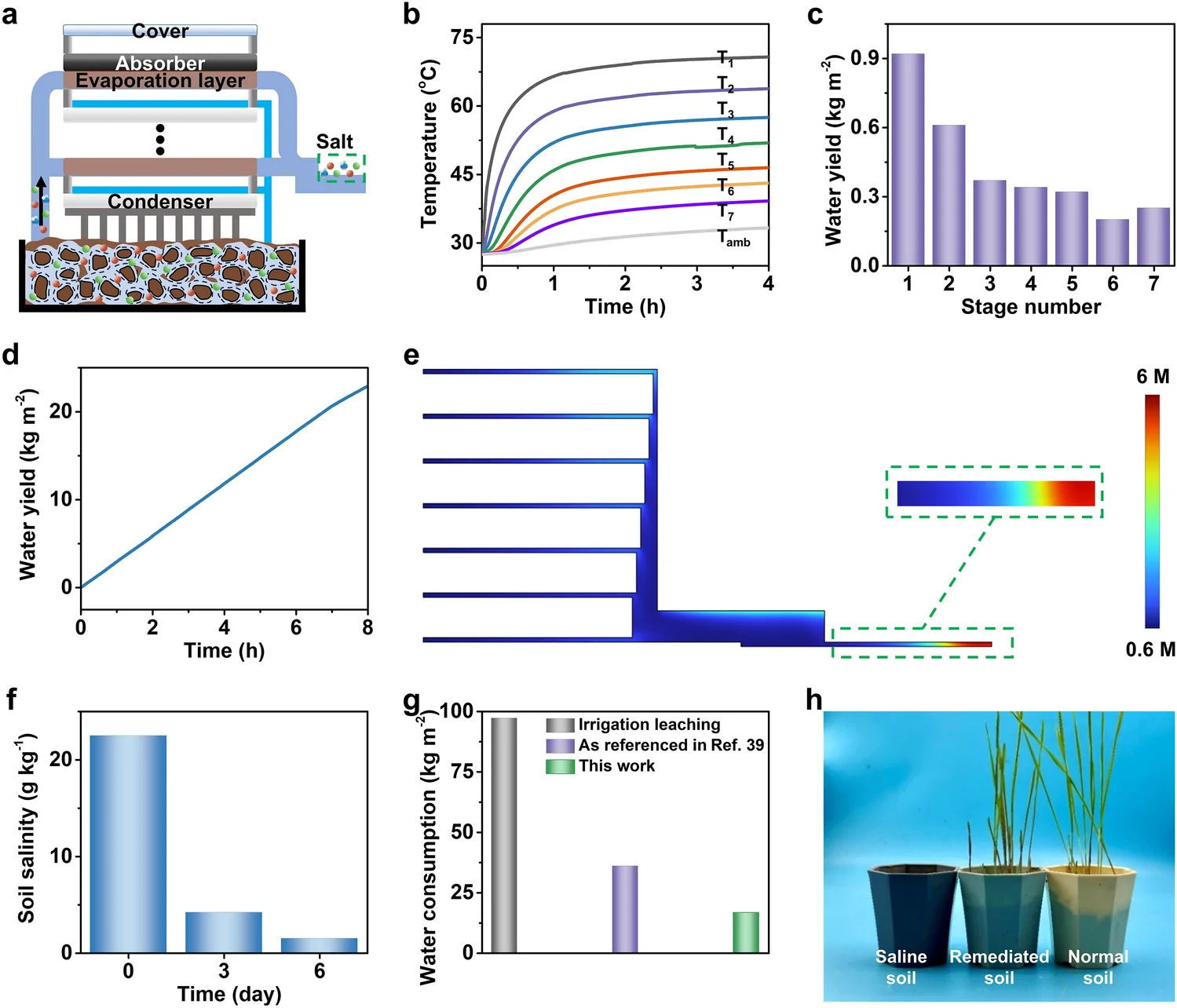
Desalination performance of the multistage solar distiller. **a** Schematic diagram of multistage solar distiller for soil desalination. **b** Temperature of every evaporation layer under one-sun irradiation during saline soil desalination. **c** Individual water yield of every stage of the solar distiller. **d** Water yield of the multistage solar distiller during saline soil desalination. **e** Simulated salinity distribution in the fabric. **f** Soil salinity during remediation process. **g** Water consumption for soil desalination using irrigation leaching, interfacial solar evaporator, and multistage solar distiller. **h** Crop growing in normal soil, remediated saline soil, and untreated saline soil

To further enhance the desalination ratio of the model-III device, a multistage solar distiller and is constructed to reuse the latent heat (Note S4 and Figs. S21 and S22) of water evaporation and enhance the water yield. An increased water production rate of the distiller enables a greater volume of water to be recycled for soil leaching (Fig. 4a), thereby improving desalination efficiency. The multistage solar distiller was first tested in a 0.6 M NaCl solution with an excess water supply. Under one-sun irradiation, the temperature of the absorber stabilized at approximately 68 °C after 1 h (Fig. S23), achieving a water yield of 3.6 kg m^−2^ h^−1^ and a solar-steam efficiency of 235%. Subsequently, the multistage solar distiller was used for saline soil desalination (Fig. S24). A replaceable fabric was incorporated beneath the extended zone of the solar distiller to serve as the salt collection layer (Figs. S25 and S26). The evaporation layer of each stage and the replaceable fabric was cut into small pieces with the wide of 1 cm after 8 h of operation on saline soil the salt concentration of each piece was measured. As shown in Fig. S27, the presence of replaceable fabric ensures the evaporation zone keep in low concentration. The collected water is recycled and directly infiltrated into the soil, eliminating the external water supply. Under one-sun irradiation, the temperature of the absorber is about 70 °C after 1 h (Fig. 4b). The individual water yield of each stage is shown in Fig. 4c. The total water yield is about 3 kg m^−2^ h^−1^ over 8 h (Figs. 4d and S28), with a solar-steam efficiency of 200%. The produced water exhibited very low salinity, meeting the WHO drinking water standards (Fig. S29). We constructed an evaporation model for the multistage solar distiller (Fig. S30), simulating the salinity distribution across the upper nonwoven fabric in the distiller. The simulation process is detailed in Note S5, with parameters specified in Fig. S31 and Tables S1 and S5. As shown in Fig. 4e, salt preferentially accumulates in the replaceable layer, thereby enabling the maintenance of low salt concentration in the fabric within the distiller through periodic replacement of the fabric. So, this replaceable layer is replaced every day following, and after six days of continuous operation, the soil salinity decreased from 22.5 to 1.49 g kg^−1^ (Fig. 4f), reaching the level of lightly saline soil (Table S7). The total water consumption was only 16.7 kg m^−2^, much smaller than other methods (Fig. 4g and Table S8). We have also compared the cost and energy consumption among the three technologies (Note S7 and Table S9), and the results reveal that multistage solar distiller exhibits the lowest total consumption, verifying its promising application prospects in practical scenarios. Then the desalinated saline soil was used to plant ryegrass. As shown in Fig. 4h, ryegrass grows normally in remediated soil, comparable to that in normal soil, whereas no plant growth was observed in the untreated saline soil. These results demonstrate that the multistage solar distiller enables stable and efficient salt extraction with minimal water consumption.

**Fig. 5:**
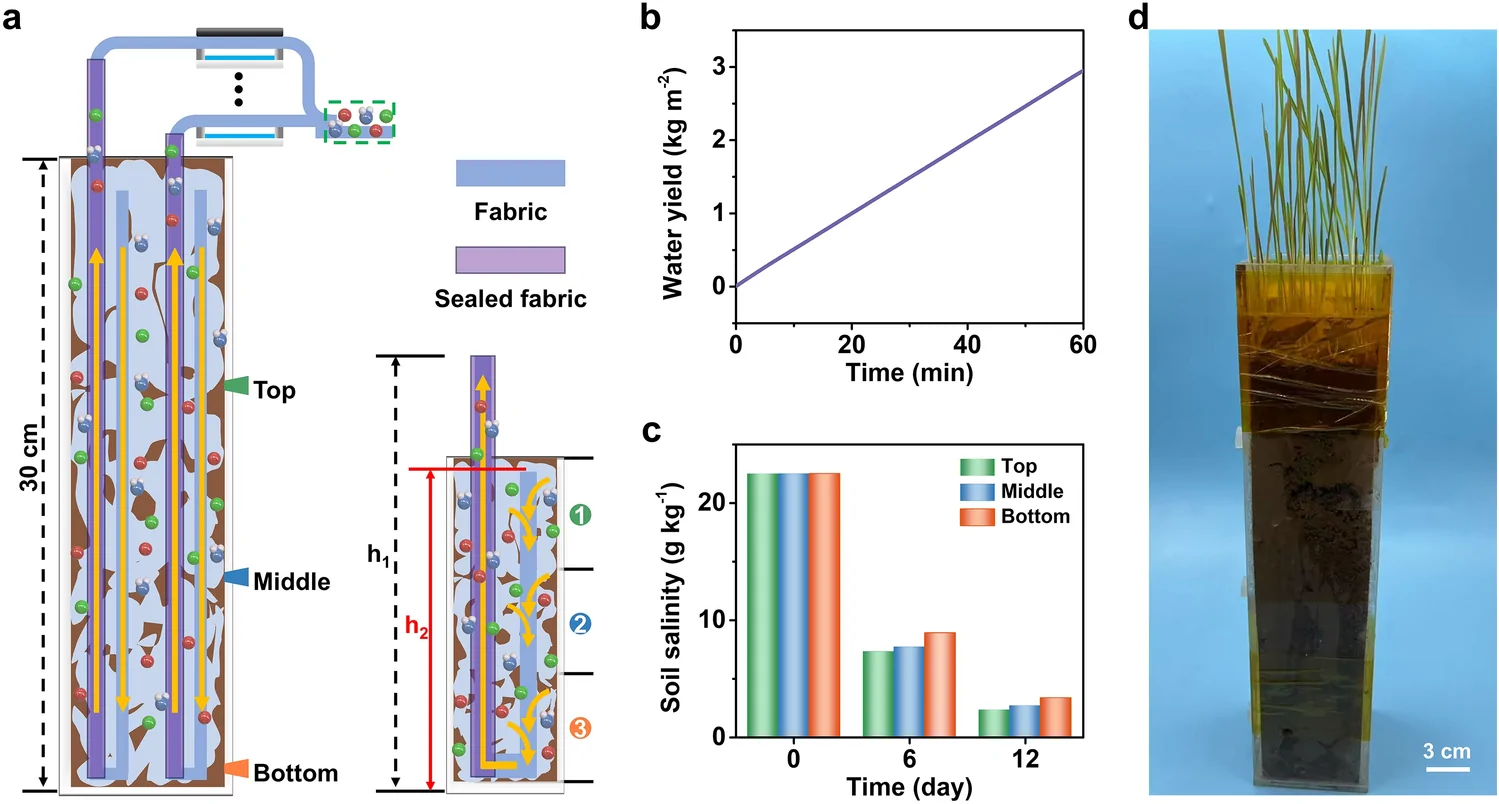
Remediating thick saline soil with multistage solar distiller. **a** Schematic diagram of multistage solar distiller desalinating saline soil with a thickness of 30 cm with a U-tube to reduce the effective water absorption height. **b** Water yield of the solar distiller. **c** Soil salinity at different depths during the remediation process. **d** Crop growing in the remediated saline soil

Deep soil desalination is more challenging than shallow soil desalination. This is because when cotton fabric is inserted into the soil, the salt solution in the near-surface region is preferentially extracted by capillary action due to its lower transport resistance and less work required to overcome gravity (Fig. S32a), making it difficult to extra salt from deep soil. If the upper region of the cotton fabric near the soil surface is sealed with a polytetrafluoroethylene (PTFE) membrane while its lower end is left exposed to the soil, and the fabric is inserted into 30 cm-thick soil (Fig. S32b), the salt solution in the deep soil can be preferentially extracted by capillary. However, the salt solution fails to reach the top of the cotton fabric because the capillary pressure of the fabric (ρgh_0_) is lower than the liquid pressure (ρgh_1_) of Region 3 (Fig. S32c). The infrared characterization results of the water-absorbent cotton fabric also corroborate this conclusion (Fig. S33a). To address this issue, the cotton fabric was designed into a U-shaped configuration in the soil to enhance the water supply as shown in Fig. 5a. The left side of the U-shape fabric is sealed with a PTFE membrane, while the right side is exposed to the soil. Based on the principle of communicating vessels, the salt solution in Region 3 can be preferentially extracted by capillary action when the liquid pressure difference between the two sides of the U-shaped structure (ρg(h_1_-h_2_)) is lower than the capillary pressure of the cotton fabric (ρgh_0_). The infrared characterization results verified that this design successfully transported water from the deep region of soil to the top layer of the cotton fabric (Fig. S33b). Then a seven-stage solar stiller with this U-tube was then used to desalinate saline soil with a thickness of 30 cm. Under one-sun irradiation, the multistage solar distiller maintained a continuous freshwater yield of 3 kg m^−2^ h^−1^ over 8 h (Fig. 5b). After 12 days of continuous operation, the salt content at three different depths decreased from 22.5 to 2.33, 2.68, and 3.35 g kg^−1^, respectively (Fig. 5c), corresponding to an average desalination ratio of 87.6%. The salinity of these three desalinated samples is lower than 4 g kg^−1^, corresponding to moderately saline soil (Table S7). The average daily water consumption during this period was 40.1 kg m^−2^ (Fig. S34). The desalinated soil successfully sustained ryegrass growth (Fig. 5d), which validates that the multistage solar distiller can stably and efficiently remediate thick saline soil.

### Outdoor Large-Scale Saline Soil Desalination

**Fig. 6:**
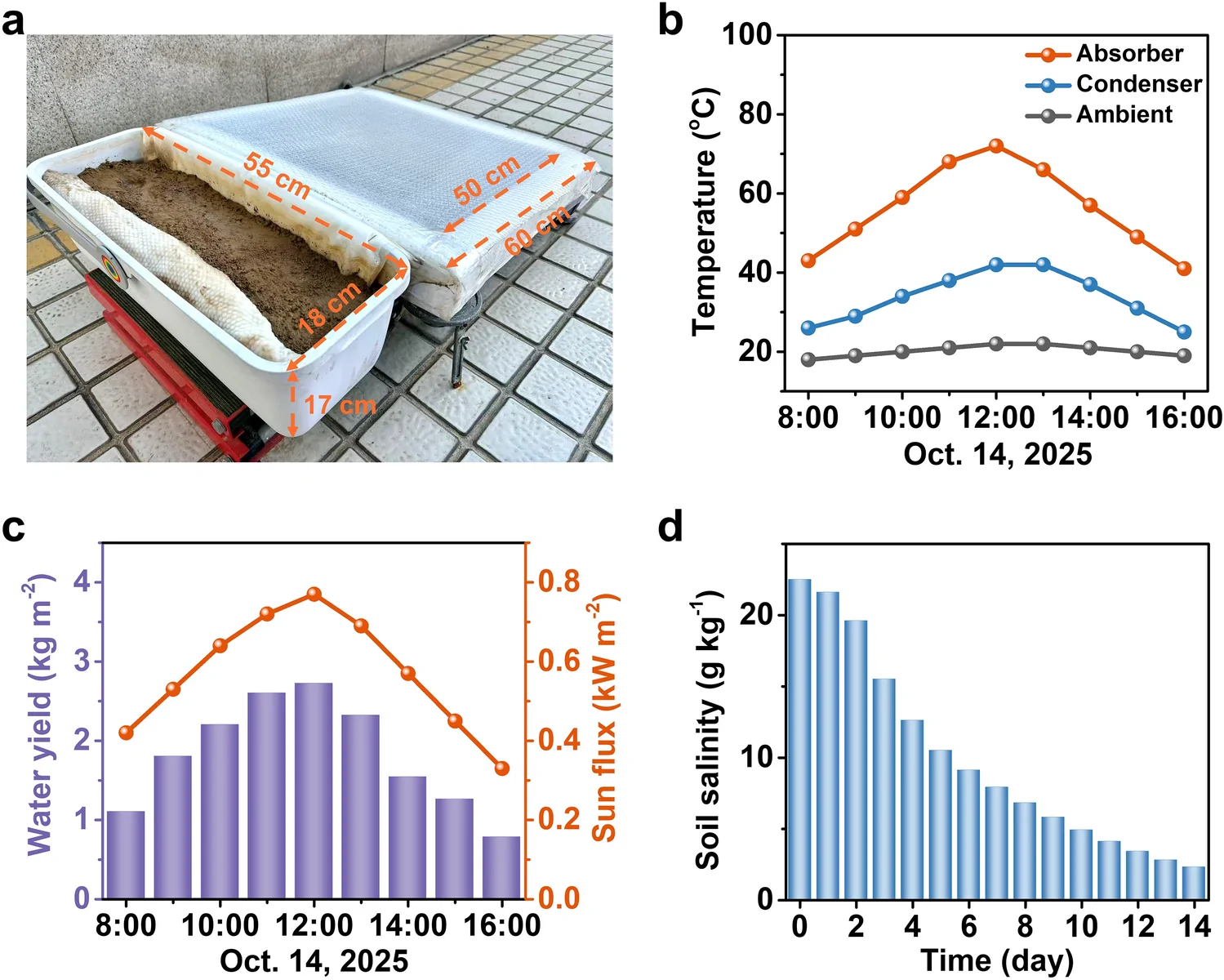
Outdoors large-scale saline soil remediation using the multi-stage solar distiller. **a** Photograph of the outdoor large-scale saline soil desalination. **b** Temperature of the absorber and condenser of the solar distiller during outdoor test. **c** Solar irradiance and water yield of the solar distiller during outdoor desalination. **d** Soil salinity of the large-scale soil during the remediation process

To evaluate practical performance under real weather conditions, a multistage solar distiller unit (50 cm × 50 cm) was used to desalinate saline soil in a large-scale (55 cm × 17 cm × 18 cm) (Fig. 6a). The device was primarily used to treat brine and verify its reliability. In the 10 h test period, the total water production reached 4225 mL, equivalent to a water yield of 17 kg m^−2^ day^−1^ (Fig. S35). Subsequently, the device was used for saline soil desalination on October 14, 2025. The temperature of the absorber reached ~ 72 °C at peak solar irradiance (0.77 kW m^−2^), with a water yield of 2.72 kg m^−2^ h^−1^ (Fig. 6b, c). The total water production was 4080 mL, equivalent to a water yield of 16.32 kg m^−2^ day^−1^. After 14 days of operation, the soil salt content was reduced from 22.5 to 2.3 g kg^−1^ (Fig. 6d), also classified as moderately saline soil (Table S7), achieving a 90% desalination ratio with an average water consumption of only 42.4 kg m^−2^ (Fig. S34). These results confirm the practical application potential of the multistage solar distiller for large-scale saline soil remediation.

## Conclusion

In summary, the developed passive multistage solar distiller presents an efficient approach for saline soil remediation. Compared with traditional open-type interfacial evaporation technologies, the multistage solar distiller enables the reusing of the latent heat of water evaporation and recycling distilled water, thereby enhancing photothermal utilization efficiency and desalination rate while reducing water consumption. Additionally, this solar still enables stable operation in large-scale treatment of deep saline soils by extending the evaporation layer fabric beyond the distiller to localize the salt-enriched zone at the edge of the evaporator and by designing a U-tube to shorten the transport height of water from deep soil to the distiller. We demonstrated the solar distiller for desalinating saline soil with a depth of 30 cm. The device achieved a freshwater collection rate of 3 kg m^−2^ h^−1^ and a solar-steam efficiency of 200%. The salt content decreases from 22.5 to 2.33 g kg^−1^ with a water consumption of 40.1 kg m^−2^. Furthermore, outdoor field testing demonstrated that we desalinated the saline soil (55 cm × 18 cm × 17 cm) to 2.3 g kg^−1^ within 14 days, consuming 42.2 kg m^−2^ of water. This work offers an avenue for high-performance saline soil remediation and may promote the application of solar energy utilization in the sustainable development of agriculture.

## Supplementary Information

Below is the link to the electronic supplementary material.

Supplementary file1 (DOCX 11739 KB)
