# Supplementary material for: Ultra-Efficient Saline Soil Remediation with Passive Multistage Solar Distiller via Water Recycling
Source: Nanomicro Lett. 2026 May 8;18:360. doi: 10.1007/s40820-026-02207-7 (PMC13156388; doi:10.1007/s40820-026-02207-7)
Supplement: Supplementary file 1 — Supplementary file1 (DOCX 11739 KB) [file 40820_2026_2207_MOESM1_ESM.docx]

Supporting Information for

**Ultra-Efficient Saline Soil Remediation with Passive Multistage Solar Distiller via Water Recycling**

Junhui Li ^1^, Jiebin Tang ^1^, Guangchun Yang ^1^, Dengchao Liang ^1^, Yi Wang ^1^, Wenwen Zhao ^1^, Xin Xia ^1^, Weijia Zhou ^1^, Yafang Zhang ^2*^, Dongjin Xin ^3*^, Guobin Xue ^1*^

^1^ Institute for Advanced Interdisciplinary Research (iAIR), School of Chemistry and Chemical Engineering, University of Jinan, Jinan 250022, P. R. China

^2^ School of Physics and Technology, University of Jinan, Jinan 250022, P. R. China

^3^ Shandong Key Laboratory of Ubiquitous Intelligent Computing, School of Information Science and Engineering, University of Jinan, Jinan 250022, P. R. China

*Corresponding authors. E-mail: [ifc_xuegb@ujn.edu.cn](mailto:ifc_xuegb@ujn.edu.cn) (Guobin Xue); [sps_zhangyf@ujn.edu.cn](mailto:sps_zhangyf@ujn.edu.cn) (Yafang Zhang); [xindj@e-wafer.cn](mailto:xindj@e-wafer.cn) (Dongjin Xin)

**Supplementary Notes**

**Note S1 Assembly and desalination process of model-I**

The evaporator in model-I is a CNT-coated nonwoven fabric (Fig. S3), attaching CNT particles form aggregates with sizes in the range of 2-5 μm (Fig. S4a). The water absorption height of CNT-coated nonwoven fabric has not decreased (Fig. S1b). Model-I enhances light absorption, achieving a weighted absorptance of 96% under the AM 1.5 G solar spectrum (Fig. S4b). The left section of the fabric is inserted into saline soil (Fig. S5) with 40% water content to extract saline solution. A horizontal section of the fabric, with an area of 5 cm × 5 cm and supported underneath by an aluminum sheet, serves as the evaporation zone. The saline solution is transported to this zone via capillary action and, after solar heating, evaporates directly into the atmosphere. Under one-sun irradiation, the upper surface temperature of model-I increased from 23°C to 40°C within 10 minutes (Fig. S6). During an 8-hour continuous desalination tests without external water addition, the evaporation rate of model-I reached 0.88 kg m^-2^ h^-1^ in the first hour (Fig. S7) but gradually decreased thereafter. This decline is attributed to the continuous loss of vapor to the environment, which progressively depletes the total water content in the system and thus reduces the supply of saline solution available for evaporation. Following the aforementioned evaporation process, the salts within the saline soil are retained within the evaporator. We constructed an evaporation model of model-I (Fig. S8), simulating the salinity distribution across the evaporator using COMSOL. The simulation process is detailed in Note S5, with parameters specified in Fig. S9 and Tables S1, S2. Furthermore, salinity distribution (Fig. S10a), and salt crystallization images (Fig. S10b) indicate salt accumulation within the evaporation zone of model-I. This accumulation blocks the capillary channels, thereby impeding saline solution transport to the evaporation zone and lowering the evaporation rate. Additionally, salt deposition hinders light absorption on the fabric surface, compromising the photothermal efficiency of the CNT coating.

**Note S2 Assembly and desalination process of model-II**

In order to collect the generated vapor as freshwater for soil irrigation—thereby maintaining soil moisture and reducing the demand for external water supply—we developed model-II (Fig. S11), a single-stage solar distiller without capillary layer extension. Model-II is based on the nonwoven fabric but features a sealed evaporation zone, maintaining the same area of 5 cm × 5 cm. It employs a solar absorber as the photothermal material, which is in contact with the upper surface of the nonwoven fabric. Beneath the fabric are an air gap and a condenser layer. The left section of model-II is inserted into soil with 40% water content (Fig. S12) to draw saline solution, which is transported to the evaporation zone and heated by the blue film. The water evaporates, and the vapor diffuses downward through the gap, condensing into liquid water on the condenser layer. The condenser is then collected via a conduit or directed to irrigate the soil. Under 1-sun irradiation, the temperature distribution of model-II (Fig. S13) shows the evaporation layer reaching 63°C and the condenser layer reaching 41°C within 20 minutes. The water production rate of model-II reached 0.925 kg m^-2^ h^-1^ (Fig. S14). We constructed an evaporation model of model-II (Fig. S15), simulating the salinity distribution across the evaporator using COMSOL. The simulation process is detailed in Note S5, with parameters specified in Fig. S9 and Tables S1, S3. Furthermore, salinity distribution (Fig. S10a) and salt crystallization images (Fig. S10c) reveal that salt accumulates within the evaporation zone of model-II. This accumulation clogs the capillaries, hindering saline solution transport and leading to reduced water production. Moreover, salt accumulation in the evaporation zone can increase the salinity of the produced water. Therefore, model-II cannot achieve stable evaporation and desalination.

**Note S3 Assembly and desalination process of model-III**

To relocate salt accumulation away from the evaporation zone, we developed model-III, the single-stage solar distiller with capillary layer extension (Fig. S16). Based on model-II, model-III retains the same evaporation zone area (5 cm × 5 cm) but extends the nonwoven fabric beyond this zone by an additional area of 4 cm × 5 cm. This extended portion is termed the "extended zone". The configuration of the evaporation zone in model-III is identical to model-II, utilizing the absorber as the photothermal material in contact with the fabric, with an air gap and condenser layer beneath. The left section is inserted into soil with 40% water content (Fig. S17) for solution uptake, which is heated in the evaporation zone. The vapor diffuses downward, condenses on the condenser layer, and the condenser is collected or used for irrigation. Under one-sun irradiation, model-III achieved a water production rate of 0.89 kg m^-2^ h^-1^ (Fig. S18a). The evaporation rate of "extended zone" is 0.335 kg m^-2^ h^-1^ (Fig. S18b). During continuous 8-hour operation without external water addition, the water production rate of model-III remained stable (Fig. S19). We constructed an evaporation model of model-III (Fig. S20), simulating the salinity distribution across the evaporator using COMSOL. The simulation process is detailed in Note S5, with parameters specified in Fig. S9 and Tables S1, S4. Furthermore, salinity distribution (Fig. S10a), and salt crystallization images (Fig. S10d) confirm that salt primarily accumulates in the extended zone, while the evaporation zone maintains very low salinity. Compared to model-I, model-II and model-III exhibits a stable evaporation rate and effectively prevents salt accumulation in the critical evaporation zone, demonstrating superior operational stability for sustainable desalination.

**Note S4 Assembly and desalination process of multistage solar distiller**

We have constructed a multistage solar distiller based on the model-III. To ensure high solar-thermal conversion efficiency while avoiding the additional cost and complexity of excessive stages. We established a model of heat and mass transfer (Fig. S21a) and an iterative algorithm (Fig. S21b). The simulation calculation process is shown in Note S6, then we calculated the water production rate and efficiency under different stage configuration (Fig. S22). Ultimately, we selected the seven-stage solar distiller.

As the solution from saline soil contains various ions, a difficult-to-separate salt crust will form in the fabric (Fig. S25). The crust may intrude into the sealed evaporation zone after prolonged operation, damaging the stability. So, a replaceable fabric was used at the end of the evaporator, allowing salt to crystallize and accumulate preferentially (Fig. S26). This fabric is replaced every day to collect mixed crystalloid salts and maintain the low salinity of the fabric in the sealed evaporation zone.

**Note S5 Simulation of Salinity Distribution in the Capillary Layer**

We used COMSOL Multiphysics® software to simulate the salinity distribution within the four desalination devices. For clarity, the simulation domains are not drawn to scale. The cross-section of the water transport channel can be simplified as a 2D model, as shown below for model-I, model-II, model-III, and the multistage solar distiller, respectively. For simplicity, the liquid flow field was obtained via idealized laminar flow.

Effective porosity of the evaporator. Porosity was measured by saturating the matrix with water under vacuum conditions. The calculation formula is:

|  | $\theta=\frac{m_{1}-m_{2}}{\rho\cdot V}$ | $\left( S1 \right)$ |
| --- | --- | --- |

where m_1_ and m_2_ are the weights (mg) of the matrix before and after wetting, ρ is the density of water, and V is the volume of the porous matrix.

COMSOL simulation of water transport and salinity distribution. The "Laminar Flow" and "Transport of Diluted Species in Porous Media" modules were combined to simulate salinity distribution. In "Laminar Flow," the continuity equation is written as:

|  | $\frac{\partial\rho}{\partial t}+\nabla\cdot\left( \rho u \right)=0$ | $\left( S2 \right)$ |
| --- | --- | --- |

The Navier-Stokes equation is written as:

|  | $\rho\frac{\partial u}{\partial t}+\rho\left( u\cdot\nabla\right)u=\nabla\cdot\left[ -pI+K \right]+F$ | $\left( S3 \right)$ |
| --- | --- | --- |
|  | $K=\mu(\nabla u+{(\nabla u)}^{T})-\frac{2}{3}\mu(\nabla\cdot u)I$ | $\left( S4 \right)$ |

where µ is the dynamic viscosity, u is the solution velocity, and I represents the identity matrix. The transport of diluted species is governed by the advection-diffusion equation:

|  | $R_{i}=\frac{\partial\left( \theta c \right)}{\partial t}+\nabla\cdot J+u\cdot\nabla c$ | $\left( S5 \right)$ |
| --- | --- | --- |
|  | $J=- D\nabla c$ | $\left( S6 \right)$ |

where R_i_ represents the mass source due to water evaporation, θ is the porosity, c is the salt concentration, and D is the classical diffusion coefficient. The left inlet was set as an open boundary. The initial concentration c_0_ for the entire domain was set to 0.6 mol L^-1^. Parameters used in the simulations are summarized in Fig. S9, Fig. S31 and Tables S1-S5.

**Note S6 Simulation of the calculation process for a multistage solar distiller**

The multi-stage configuration can enhance the desalination rate. To investigate the heat and mass transfer processes within the multi-stage solar distiller, we established a multistage distillation model. To simplify the analysis, the following assumptions were made. (1) The temperature of the condenser layer is assumed to be equal to that of the evaporation layer in the next stage, as these layers are very thin (only 0.2 mm) and in good thermal contact; (2) The final condenser is at the same temperature as the ambient environment. Here, T_i_ denotes the temperature of the i stage evaporator, and T_b_ denotes the temperature of the final condenser. We accounted for various heat losses, including radiation (q_rad_) and convection (q_conv_) from the front solar absorber, conduction through the air gap (q_cond,i_), sidewall heat loss for each stage (q_side,i_), conductive heat loss from the extended capillary layer (q_a,i_), and convective flow heat loss from the extended capillary layer (q_b,i_). The energy balance can be expressed as:

For the first stage (i=1):

| $a^{2}q_{\mathrm{sun}}=a^{2}\left( q_{\mathrm{rad}}+q_{\mathrm{conv}}+q_{cond,i}+q_{evap,i} \right)+4abq_{side,i}+ahq_{a,i}+{a^{2}q}_{b,i}$ | $\left( S7 \right)$ |
| --- | --- |

For i stage (i>1):

|  | $a^{2}\left( q_{cond,i-1}+q_{evap,i-1} \right)=a^{2}\left( q_{cond,i}+q_{evap,i} \right)+4abq_{side,i}+ahq_{a,i}$ | $\left( S8 \right)$ |
| --- | --- | --- |

The evaporation-related heat flow is:

|  | $q_{evap,i}=h_{\mathrm{fg}}m_{evap,i}=h_{\mathrm{fg}}(-D_{a}\frac{\mathrm{dc}}{\mathrm{dX}})\approx h_{\mathrm{fg}}D_{a}\frac{c_{e}-c_{c}}{b}$ | $\left( S9 \right)$ |
| --- | --- | --- |

where m_evap,i_ is the water production rate of the i stage, h_fg_ is the enthalpy of vaporization (kJ kg**^-1^**), and D_a_ is the mass diffusivity. c_e_ and c_c_ are the saturated vapor concentrations at the evaporation and condenser layers, respectively, determined by their temperatures. The radiative heat loss from the solar absorber is given by:

|  | $q_{\mathrm{rad}}=\varepsilon\sigma\left( T_{1}^{4}-T_{\infty}^{4} \right)$ | $\left( S10 \right)$ |
| --- | --- | --- |

where ε is the emissivity, σ $=$5.67×10^−8^ W m^−2^ K^−4^ is the Stefan-Boltzmann constant, and T_∞_ is the ambient temperature. The convective heat loss from the solar absorber is:

|  | $q_{\mathrm{conv}}=h_{a}\left( T_{1}-T_{\infty} \right)$ | $\left( S11 \right)$ |
| --- | --- | --- |

where h_a_ is the convective heat transfer coefficient of air. The heat conduction through the air gap is:

|  | $q_{cond,i}=-k_{a}\frac{\mathrm{dT}}{\mathrm{dX}}\approx k_{a}\frac{T_{i}-T_{i+1}}{b}$ | $\left( S12 \right)$ |
| --- | --- | --- |

where k_a_ is the thermal conductivity of air. The heat loss through the sidewalls can be estimated as:

|  | $q_{side,i}=\frac{\bar{T}_{i}-T_{\infty}}{R_{\mathrm{side}}}$ | $\left( S13 \right)$ |
| --- | --- | --- |

where $\bar{T}$ is the average sidewall temperature, $\bar{T}$ can be calculated as:

|  | $\bar{T}=\frac{T_{i}{+T}_{i+1}}{2}$ | $\left( S14 \right)$ |
| --- | --- | --- |

The losses from the extended capillary layer are:

|  | $q_{a,i}+q_{b,i}$ | $\left( S15 \right)$ |
| --- | --- | --- |

where q_a,i_ is the conductive heat loss from the extended capillary layer, and q_b,I_ is the convective flow heat loss from the extended capillary layer. The conductive heat loss from the extension can be estimated as:

|  | $q_{a,i}=\frac{T_{i}-T_{\infty}}{R_{\mathrm{add}}}$ | $\left( S16 \right)$ |
| --- | --- | --- |

where k_w_ is the thermal conductivity of water. The convective flow heat loss from the extended capillary layer is:

|  | $q_{b}=C_{\mathrm{water}}\times m_{outlet,8}\times\left（ T_{1}-T_{\infty} \right）$ | $\left( S17 \right)$ |
| --- | --- | --- |

where m_outlet,8_ is the evaporation rate from outlet 8, C_w_ is the specific heat capacity of water.

**Note S7 Cost and energy consumption analysis of soil desalination using multistage solar distiller, interfacial evaporator and irrigation leaching**

Device cost of three technologies. Taking the remediation of saline land with an area of 100 m^2^ and a soil layer thickness of 5 cm as the calculation benchmark, the equipment and material consumption of each technology are calculated respectively. The irrigation leaching method requires a water pump and 100 m of pipelines. The materials required for the interfacial evaporator include 50 m^2^ of fabric, 0.06 kg of CNT, 6.6 L of acrylic acid and 9.2 L of 10 mol L^-1^ NaOH. The materials required for the multistage solar distiller include 50 m^2^ of photothermal materials, 350 m^2^ of fabric, 105 m^2^ of foam and 350 m^2^ of aluminum sheets. The raw material cost is estimated based on the current market prices of each component. The unit price of the water pump is $332.60, the pipeline is $0.27 m^-1^, the nonwoven fabric is $0.20 m^-2^, the CNT is $283.43 kg^-2^, the acrylic acid is $38.52 L^-1^, the NaOH is $33.87 L^-1^, the photothermal material is $0.87 m^-2^, the foam is $0.03 m^-2^ and the aluminum sheet is $0.72 m^-2^.

Operation cost of three technologies. Operation costs consist of electricity cost and water cost. The water consumption per unit area of the irrigation leaching method, interfacial evaporator and multi-stage solar distiller is 97 kg m^-2^, 36 kg m^-2^ and 16.7 kg m^-2^, respectively. The power consumption of the irrigation leaching process is 19.38 kWh. The electricity price is $0.3 kWh^-1^ and the water price is $0.73 t^-1^.

Energy consumption of three technologies. Energy consumption is electric power. The power consumption for one spray operation on saline land with an area of 100 m^2^ and a soil layer thickness of 5 cm is 19.38 kWh. The other two methods are driven by solar energy, so their electric energy consumption is zero.

**Supplementary Figures and Tables**


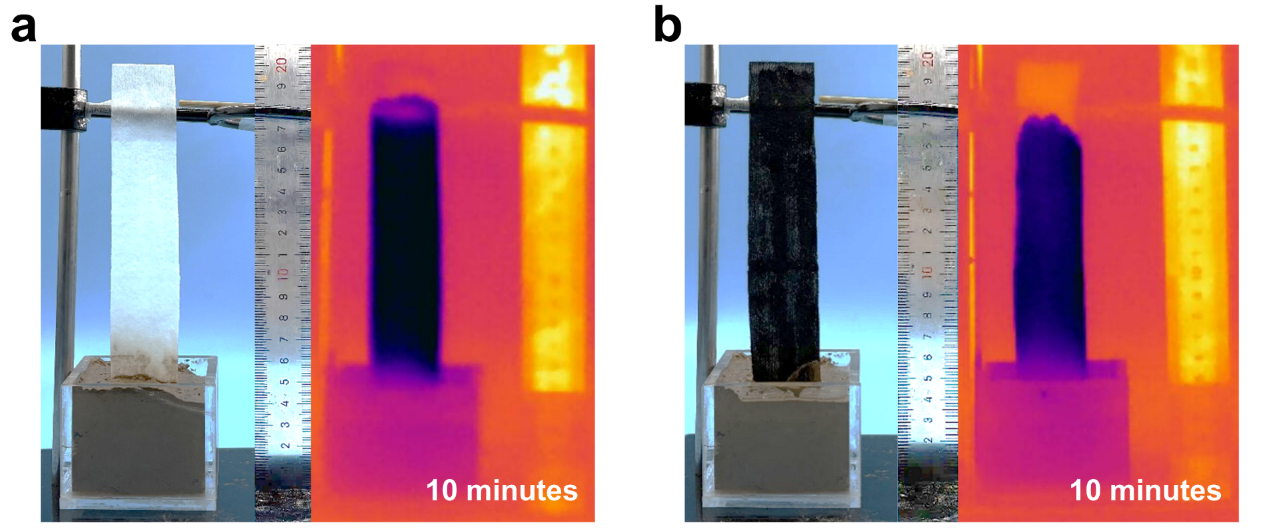


**Fig. S1** Water absorption height of the nonwoven fabric from soil with water content of 40% and the infrared image. **a** The water absorption height of the pristine (white) fabric is 13.3 cm within 10 minutes. **b** The water absorption height of the CNT-coated (black) fabric is 12.8 cm within 10 minutes.


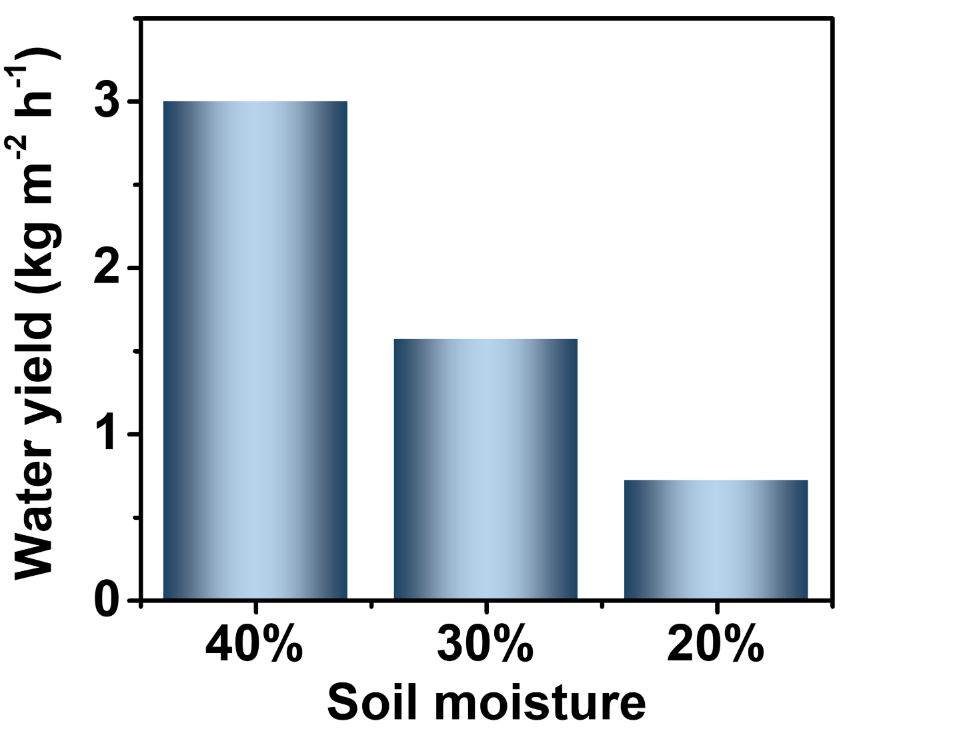


**Fig. S2** Water yield of the multistage solar distiller under different soil moisture contents.


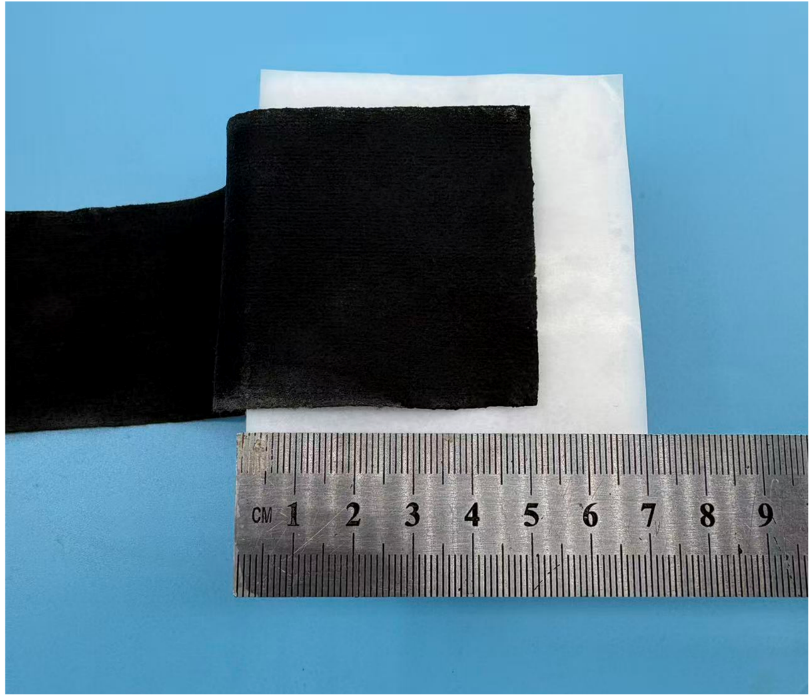


**Fig. S3** Photograph of model-I device. The CNT-coated nonwoven fabric has an evaporation area of 5 cm × 5 cm.


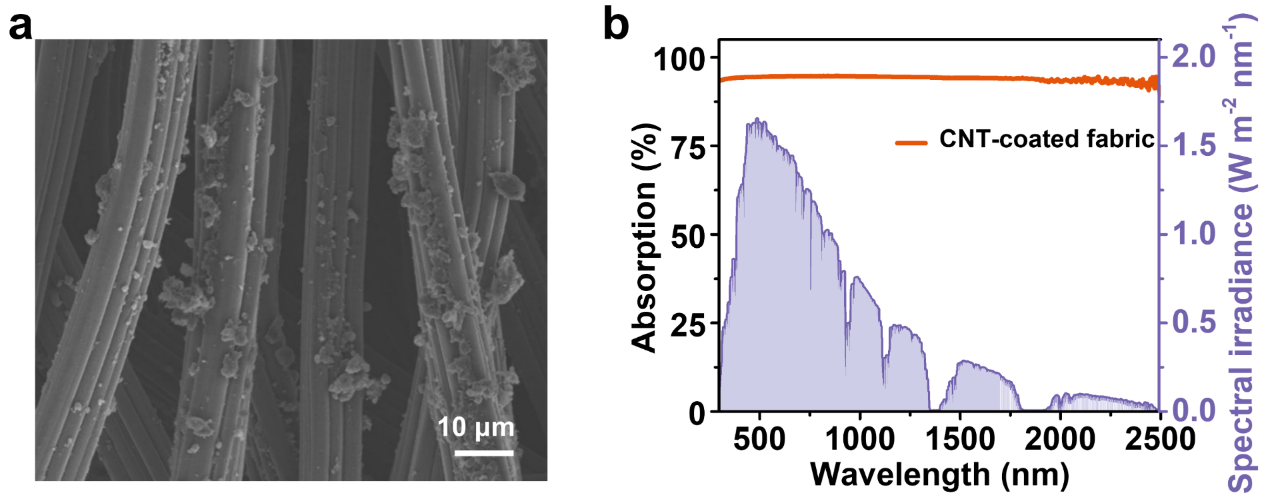


**Fig. S4** Characterization of the CNT-coated nonwoven fabric. **a** SEM image of the CNT-coated nonwoven fabric. **b** Absorption spectrum of the CNT-coated nonwoven fabric across the 300-2500 nm wavelength range under the AM 1.5 G solar spectrum.


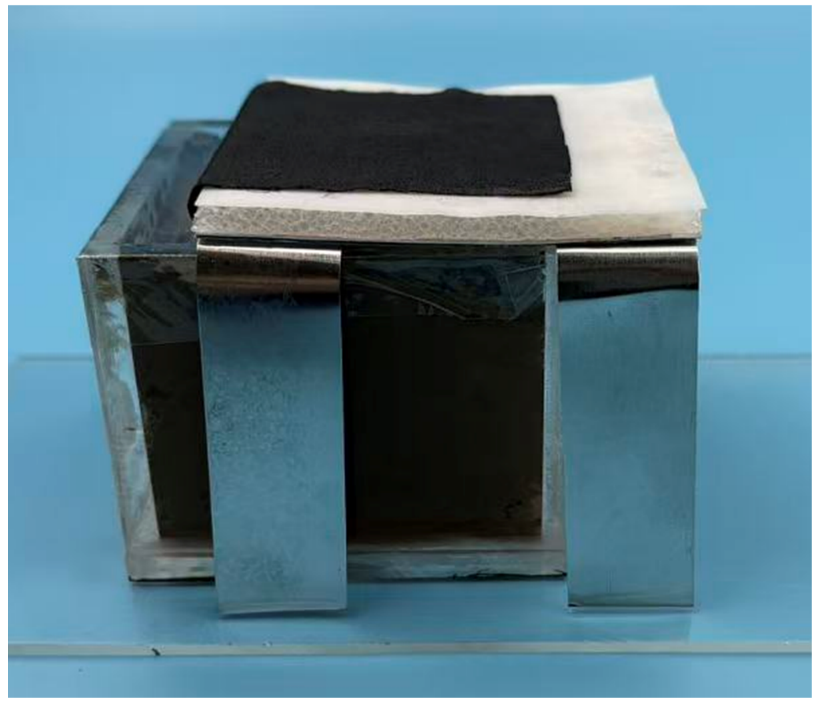


**Fig. S5** Photograph of model-I device for laboratory-scale soil desalination. The left end of the fabric is inserted into soil with a water content of 40% to absorb saline solution. The evaporation zone is insulated from the underlying soil with a polystyrene foam layer.


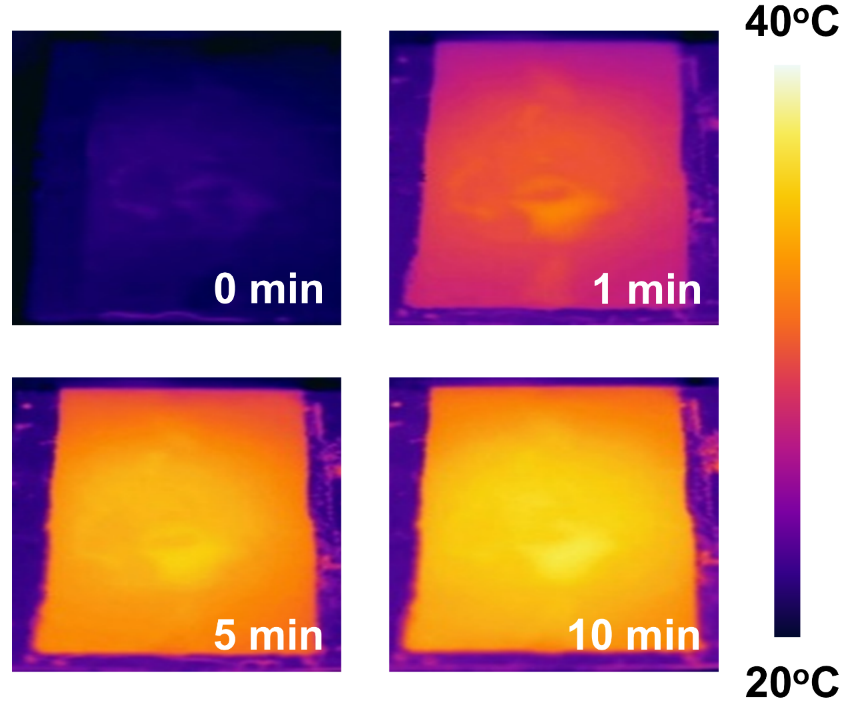


**Fig. S6** Temperature and infrared images of the evaporation zone in model-I during laboratory-scale soil desalination. The infrared images show that the temperature of the evaporation surface reaches approximately 40°C within 10 minutes.


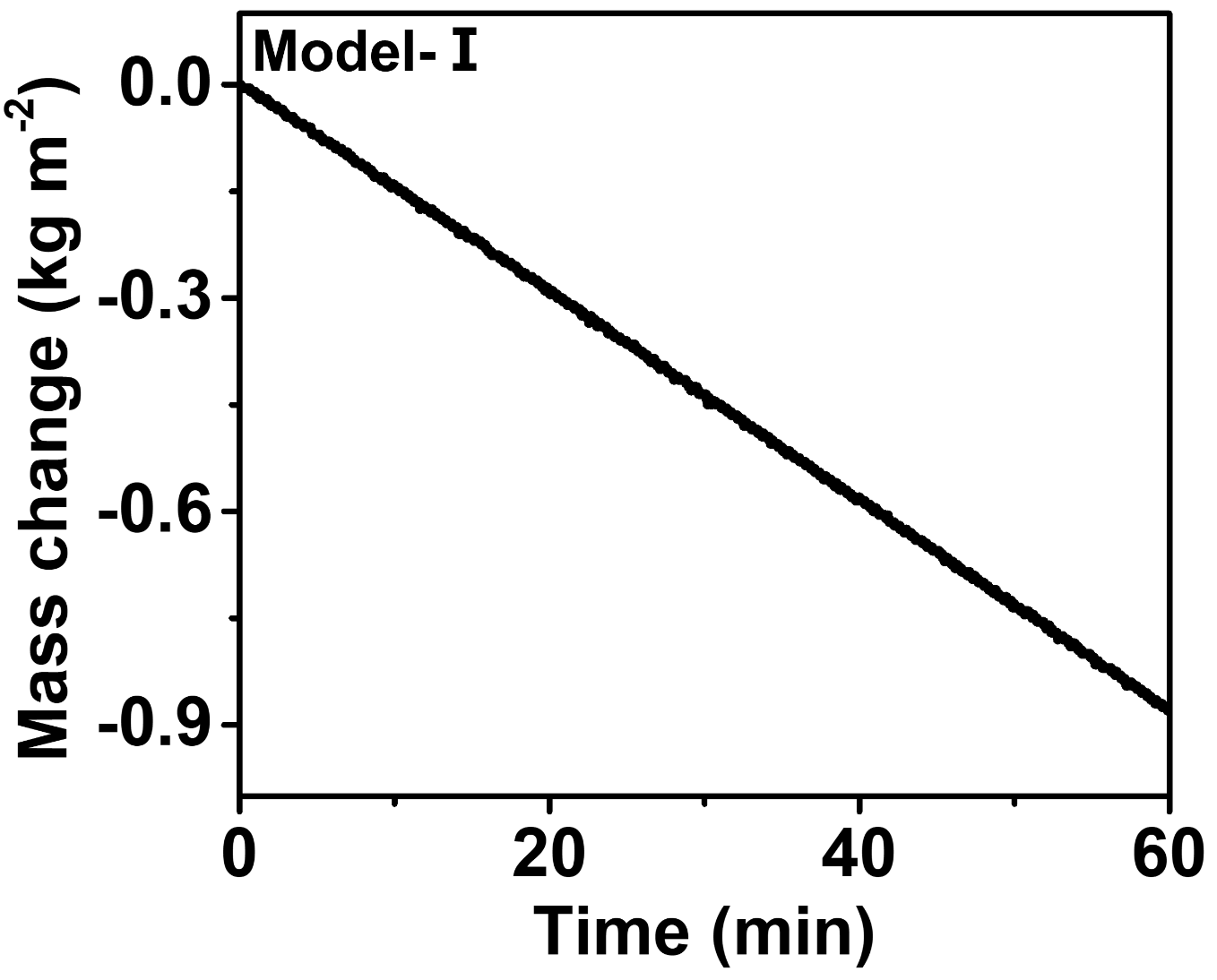


**Fig. S7** Evaporation rate of model-I device during laboratory-scale soil desalination. The evaporation rate in the first hour is 0.88 kg m^-2^ h^-1^.


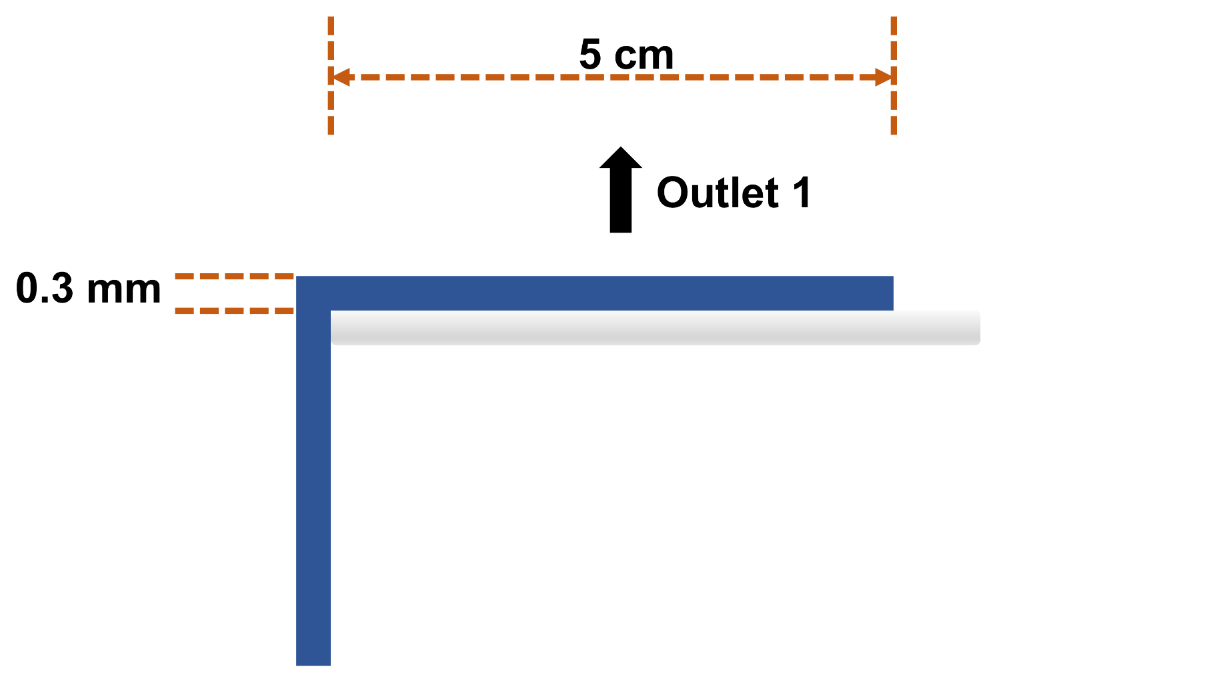


**Fig. S8** Model for COMSOL simulation of model-I device.


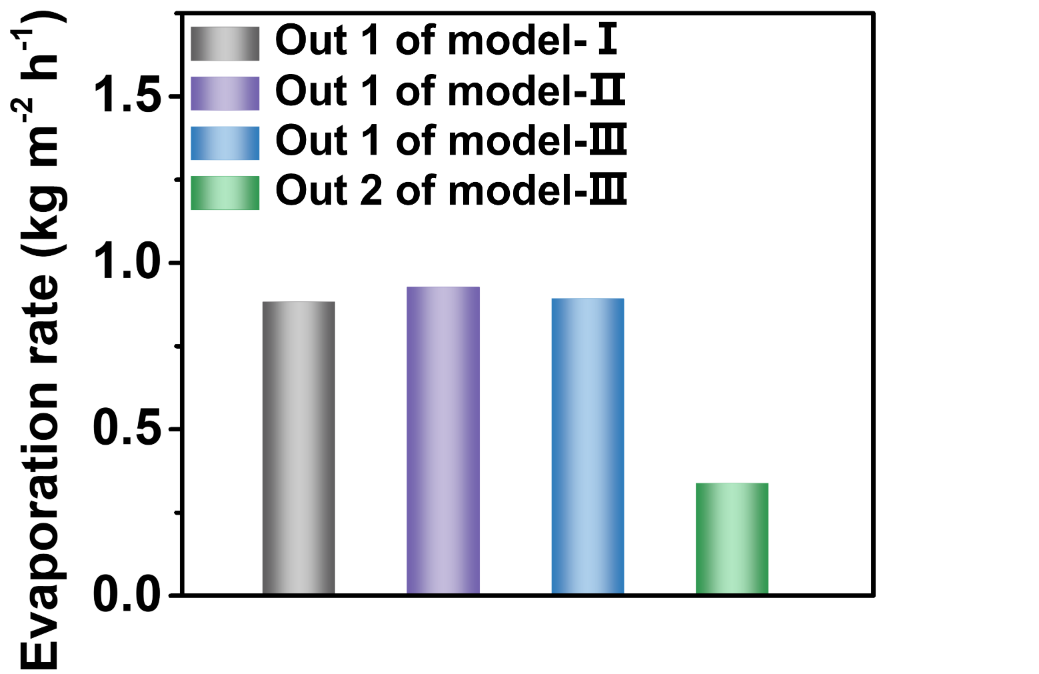


**Fig. S9** Evaporation rates of the outlet zone of model-I, model-II, and model-III, used as input parameters for COMSOL simulations.


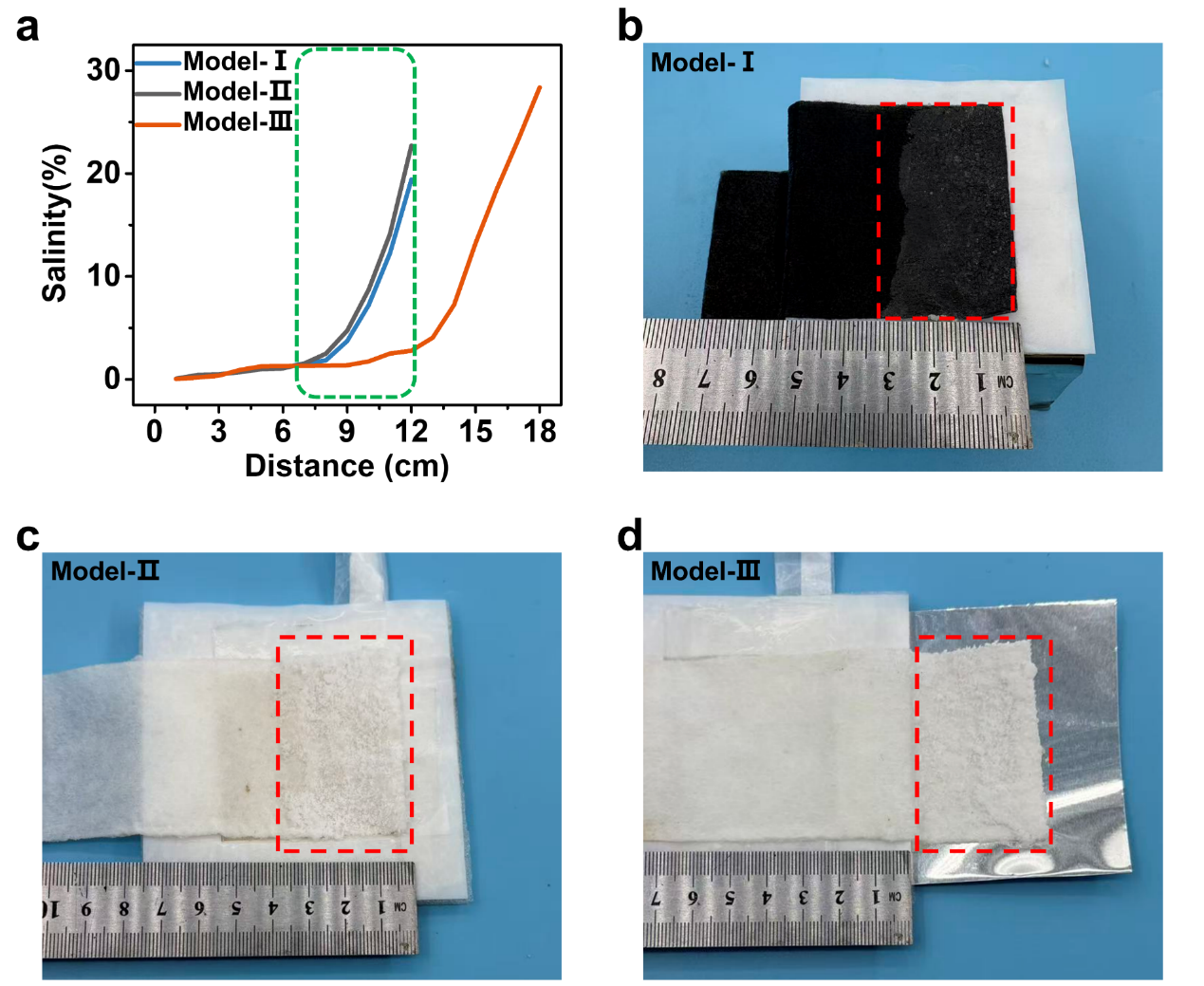


**Fig. S10** Salinity distribution in three model devices. **a** Salinity distribution for model-I, model-II, and model-III devices, respectively. The green dashed line indicates the salinity within the evaporation zone. **b-d** Photographs of salt crystallization zone for **b** model-I, **c** model-II, and **d** model-III. The red dashed line indicates the location of salt crystallization.


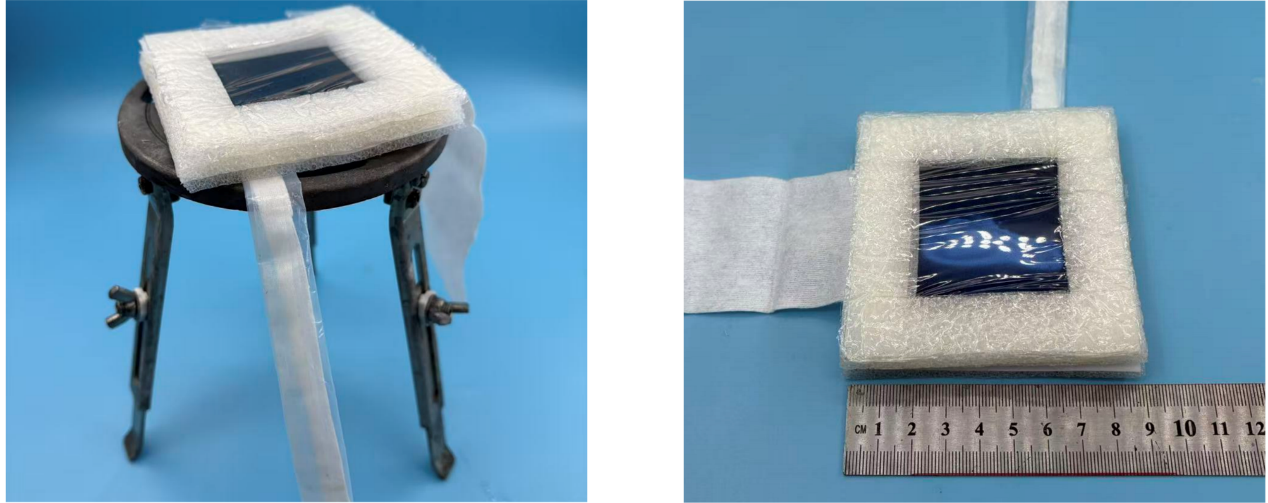


**Fig. S11** Photograph of model-II device. Model-II device is a single-stage solar distiller with the evaporation zone fully sealed. The sealed evaporation zone maintains the same area of 5 cm × 5 cm. A commercial solar absorber serves as the photothermal material, in direct contact with the upper nonwoven fabric. An air gap and a condenser layer are located beneath upper the fabric.


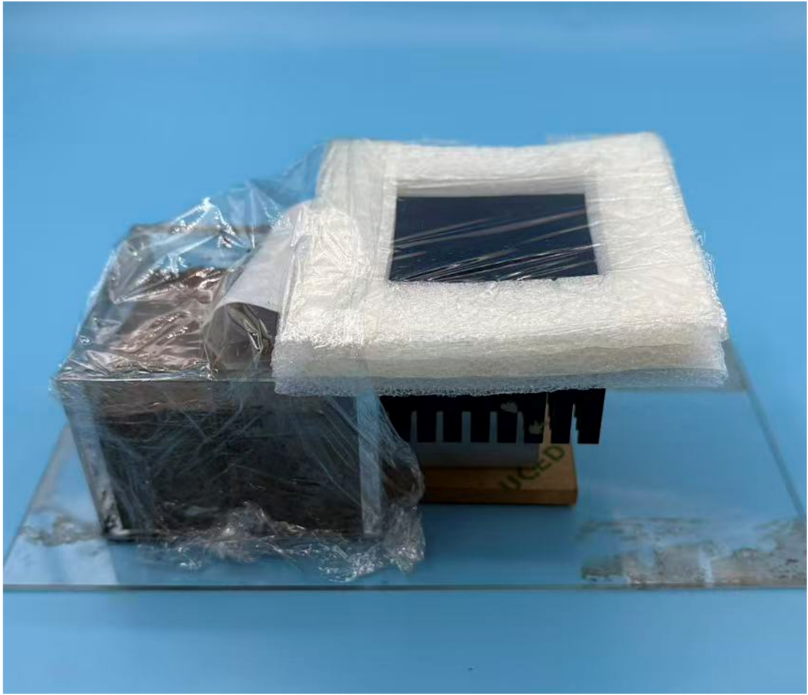


**Fig. S12** Photograph of model-II device during laboratory-scale soil desalination. The left end of the fabric is inserted into soil with a water content of 40% to absorb saline solution.


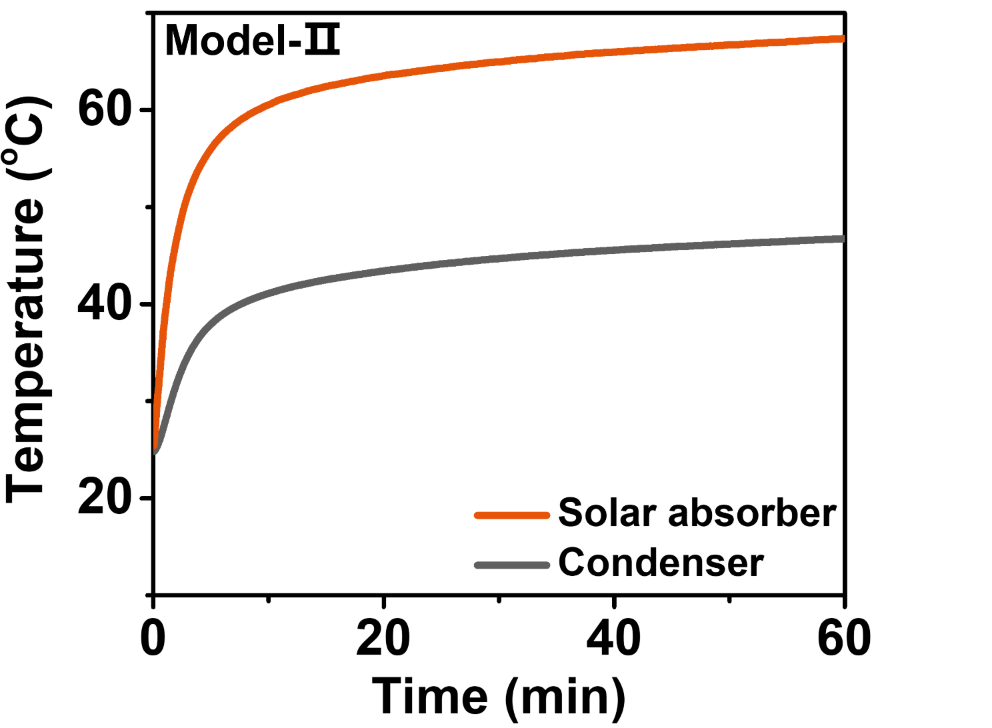


**Fig. S13** Temperature distribution of absorber and condenser in model-II device under one-sun irradiation. The temperature of the evaporation layer reaches 63°C and the condenser layer reaches 41°C within 20 minutes.


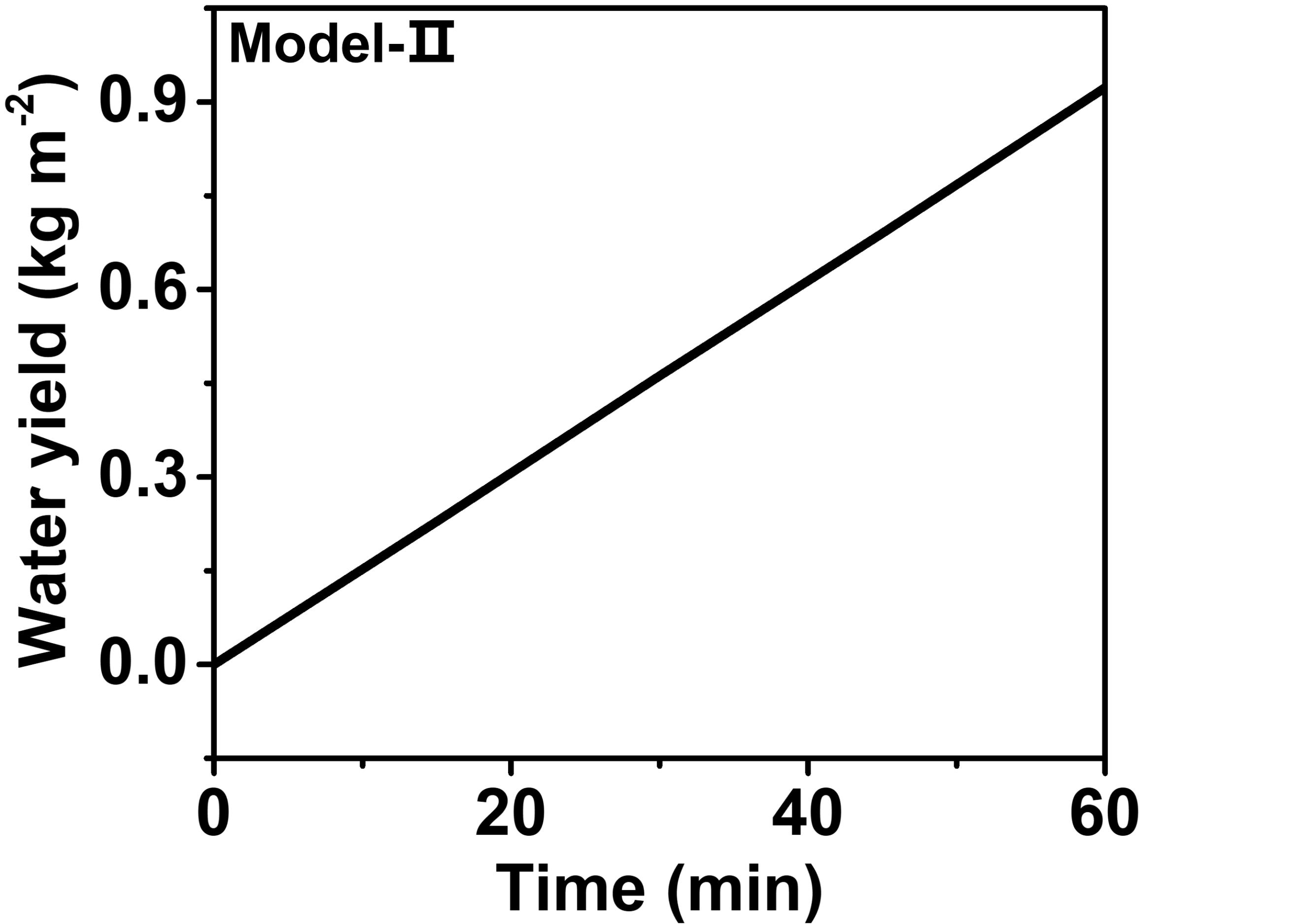


**Fig. S14** Water yield of model-II device during laboratory-scale soil desalination under one sun. The water production rate reaches 0.925 kg m^-2^ h^-1^.


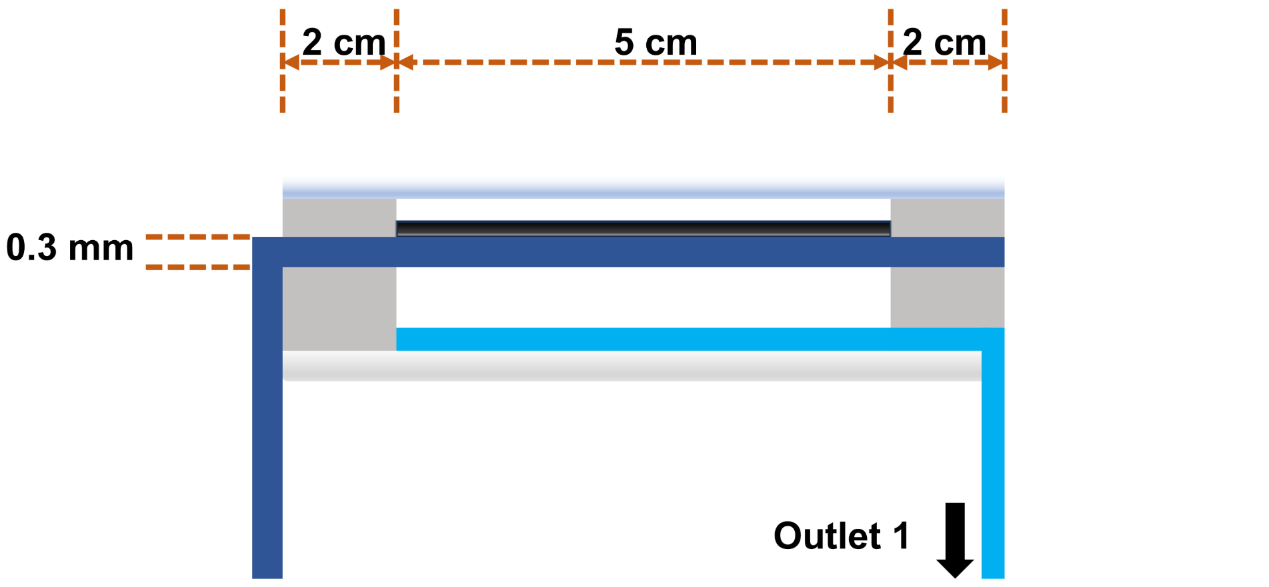


**Fig. S15** Model for COMSOL simulation of model-II device.


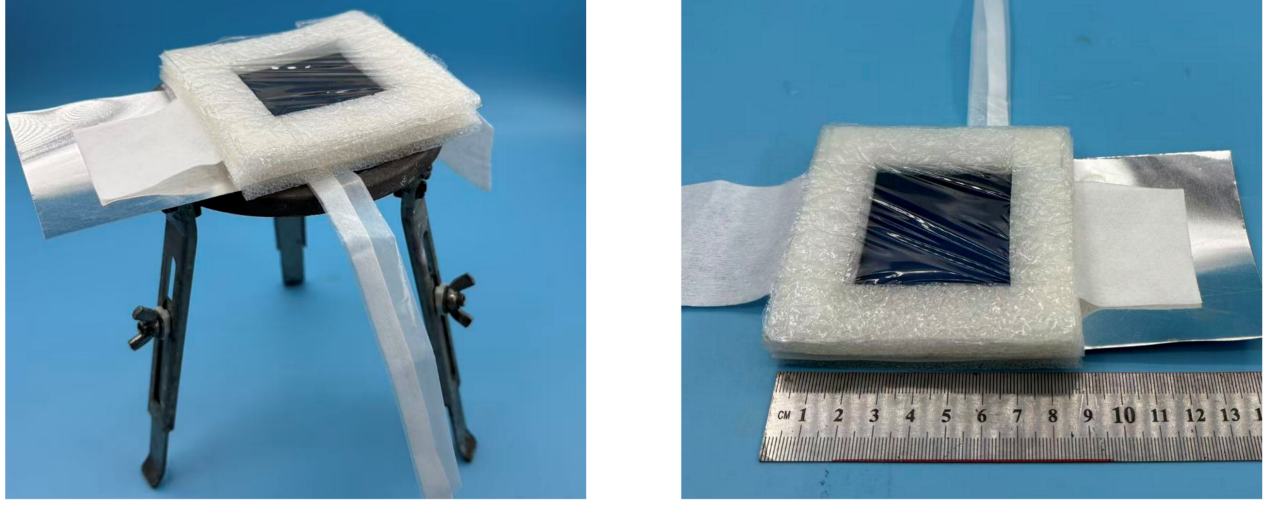


**Fig. S16** Photograph of model-III device. Model-III device is a single-stage solar distiller with the evaporation fabric extending from the distiller. The evaporation zone area in the solar distiller is 5 cm × 5 cm and the extending fabric is 4 cm × 5 cm.


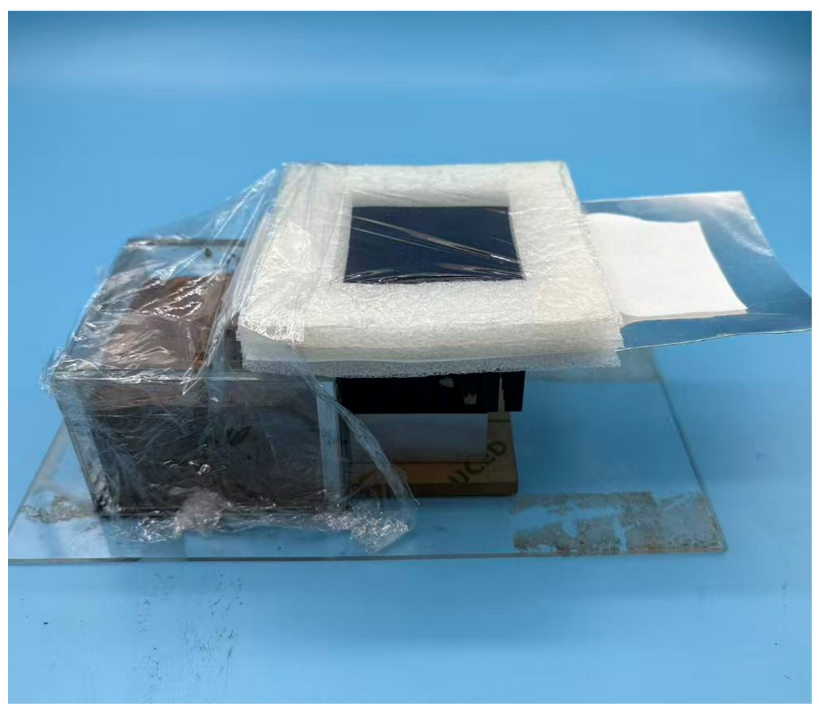


**Fig. S17** Photograph of model-III device during laboratory-scale soil desalination.


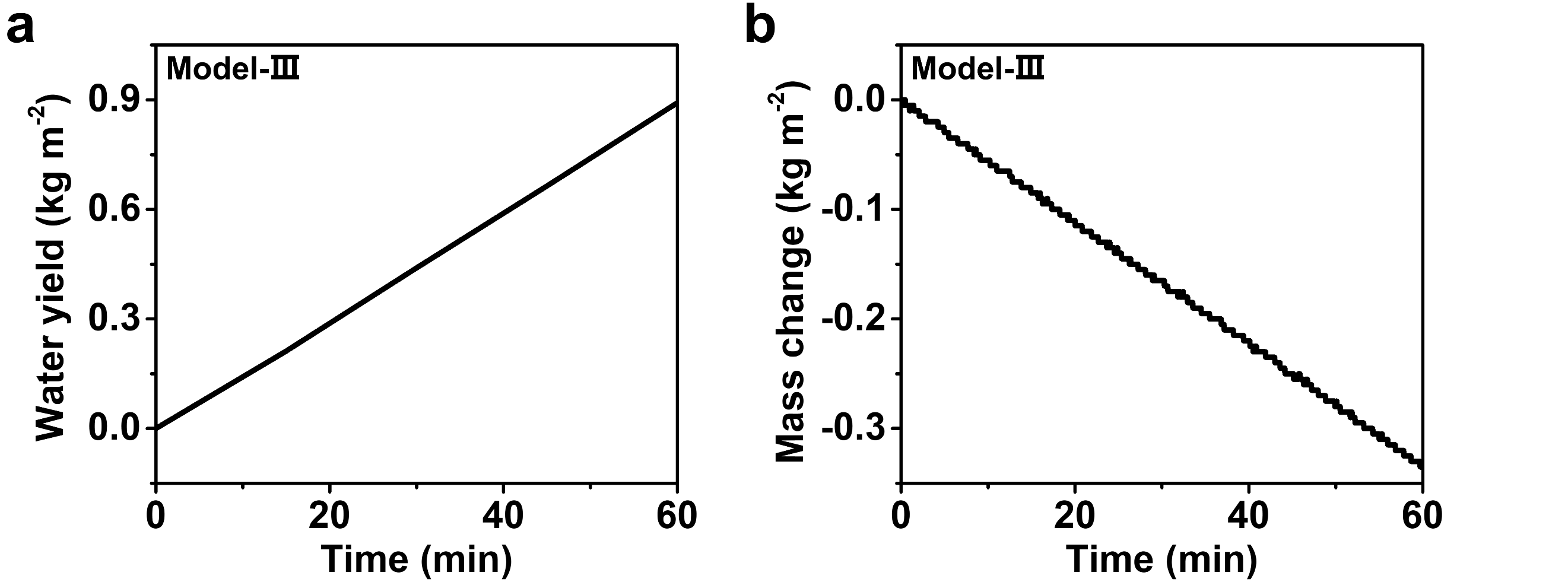


**Fig. S18** Water evaporation rate of model-III during laboratory-scale soil desalination. **a** The water production rate of model-III reaches 0.89 kg m^-2^ h^-1^. **b** The water evaporation rate of extended zone reaches 0.335 kg m^-2^ h^-1^.


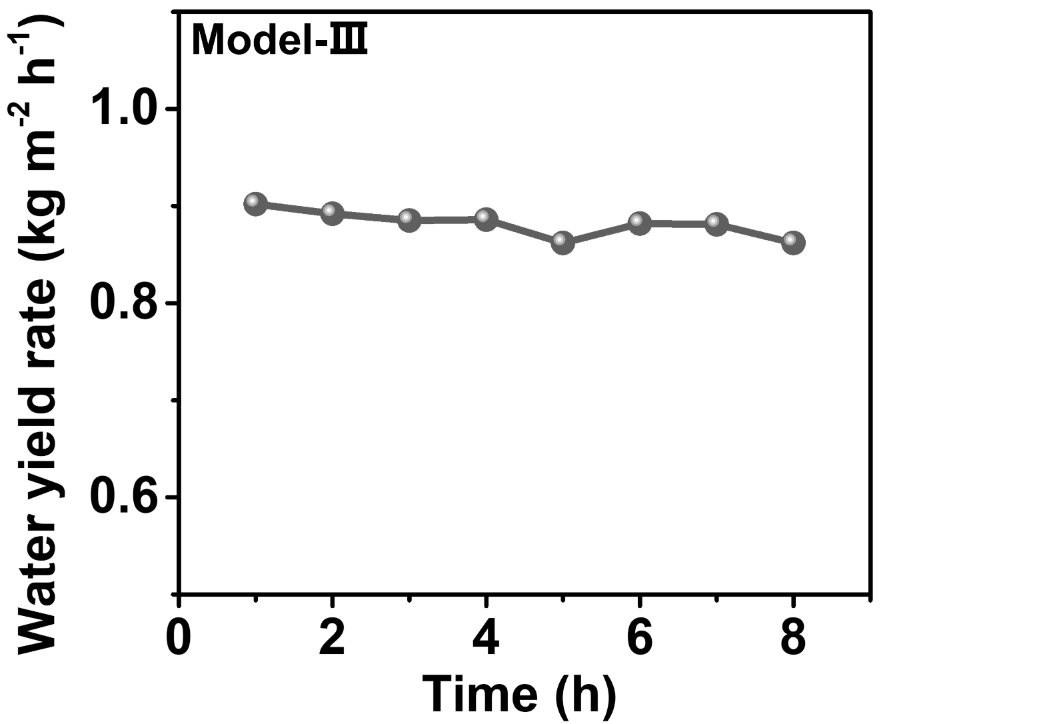


**Fig. S19** Time-dependent water yield of model-III device during laboratory-scale soil desalination. The water yield remains stable at 0.9 kg m^-2^ h^-1^ over 8 hours.


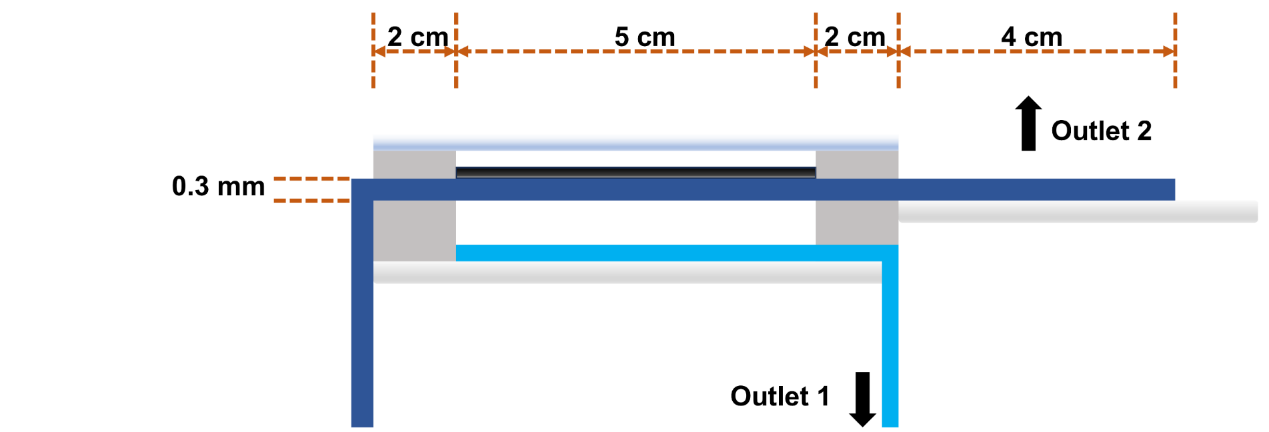


**Fig. S20** Model for COMSOL simulation of model-III device.


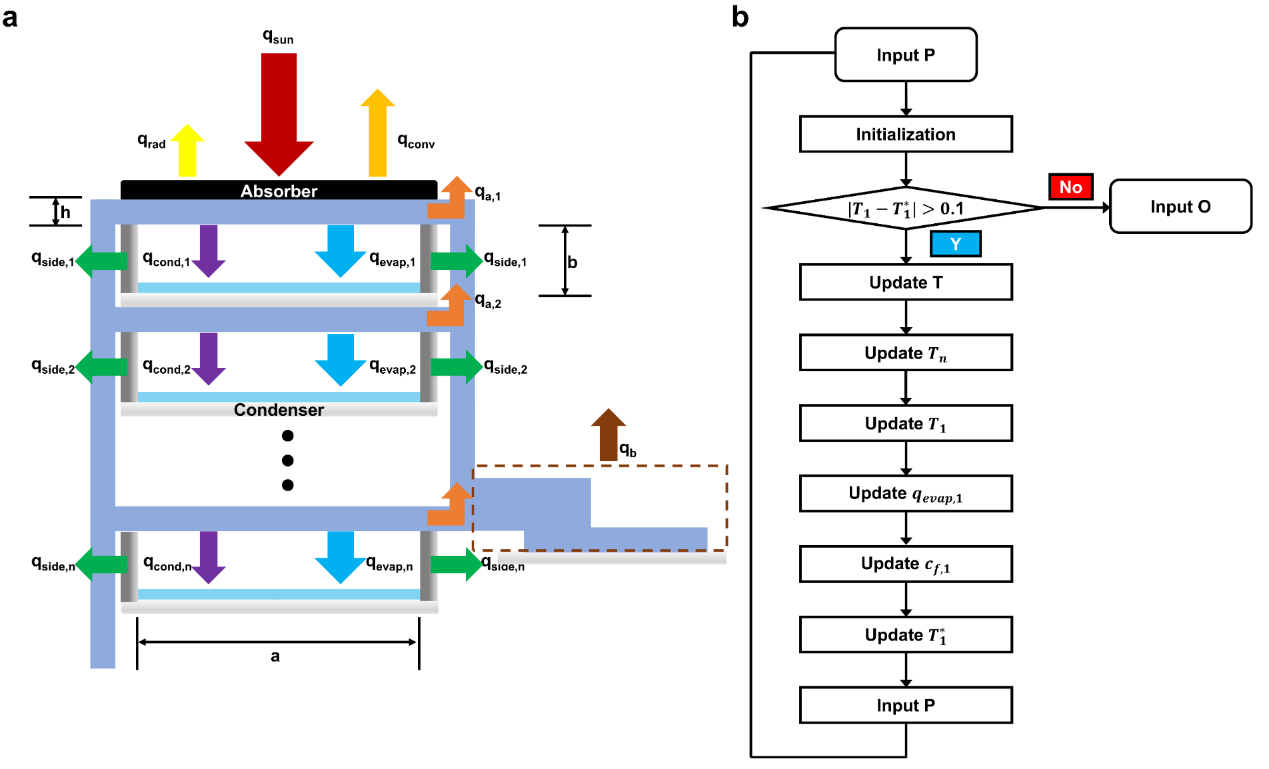


**Fig. S21** **a** Heat and mass transfer model of the multistage solar distiller. **b** Iterative algorithm for calculating the heat and mass transfer in the multi-stage solar distiller.


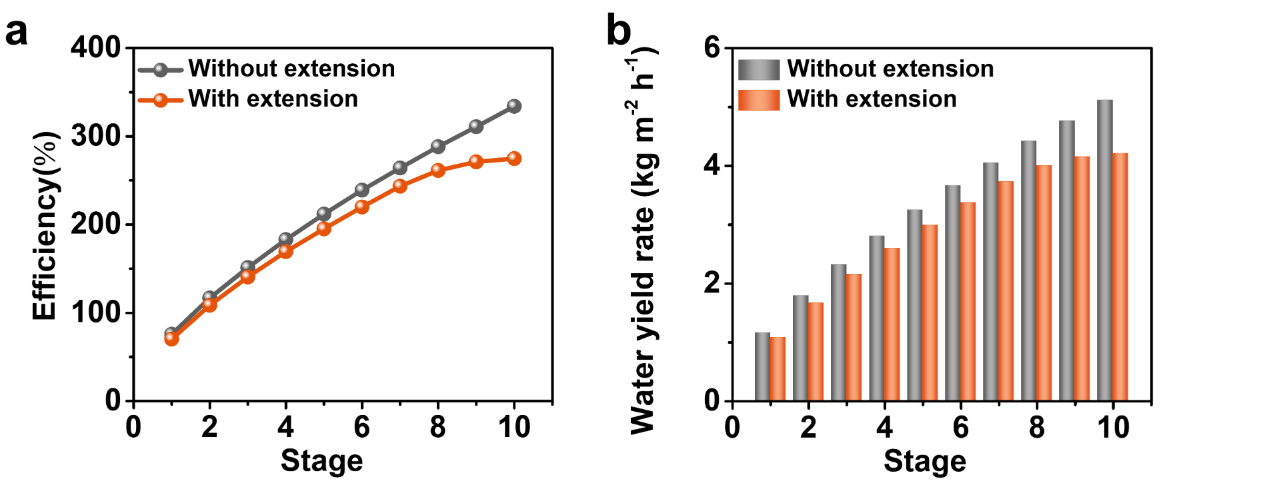


**Fig. S22** The calculated efficiency and water yield of the multistage solar distiller. **a** Efficiency of the device with different layer counts. **b** Water yield of the device with different layer counts.


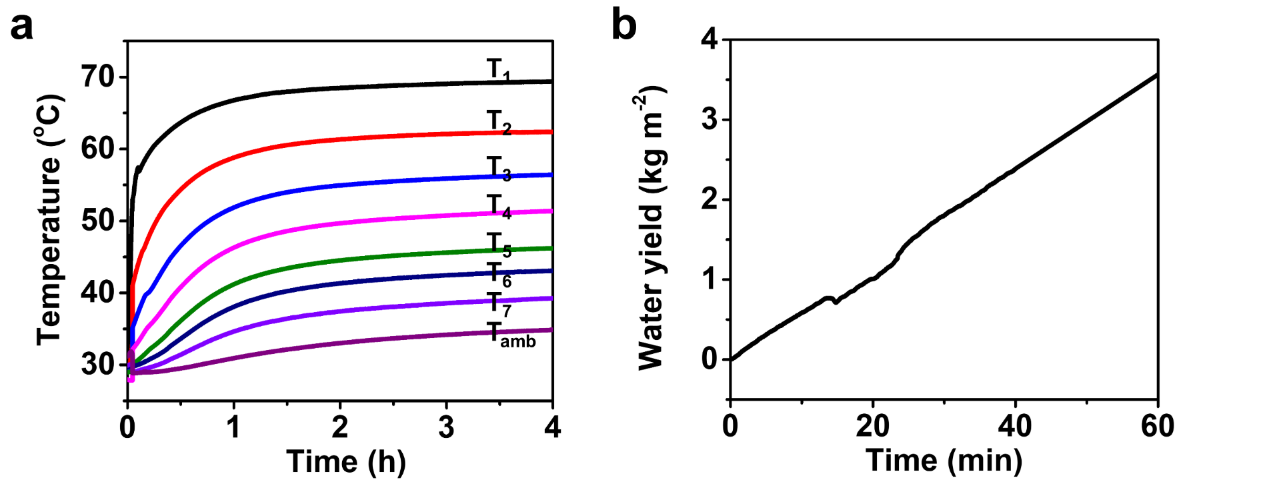


**Fig. S23** Performance of the multistage solar distiller when desalinating 0.6 M NaCl brine. (a) Under one-sun irradiation, the temperature of the evaporation layer of the first stage stabilizes at approximately 68°C after 1 hour. (b) The water yield rate is 3.6 kg m^-2^ h^-1^.


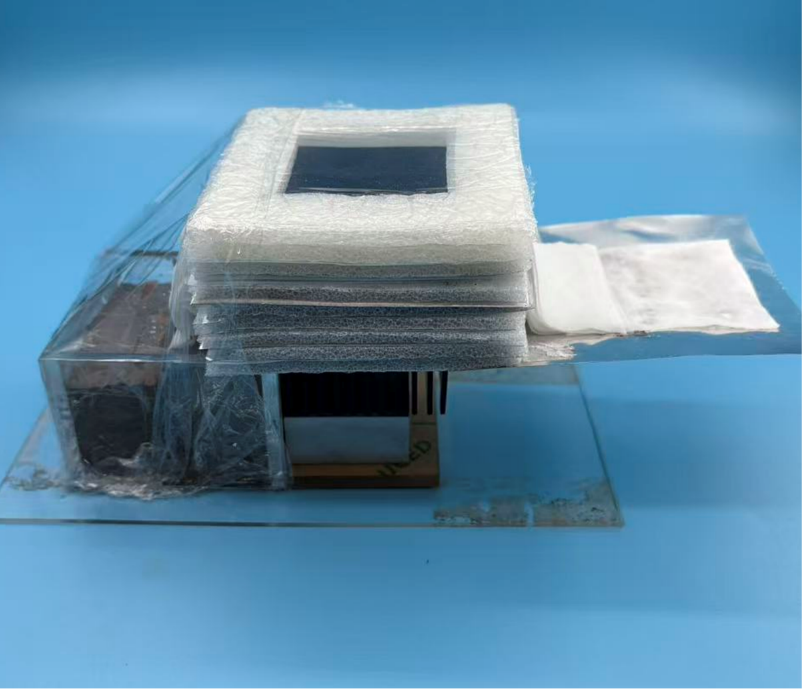


**Fig. S24** Photograph of the multistage solar distiller during laboratory-scale soil desalination. The left end of the fabric is inserted into soil with a water content of 40% to absorb saline solution.


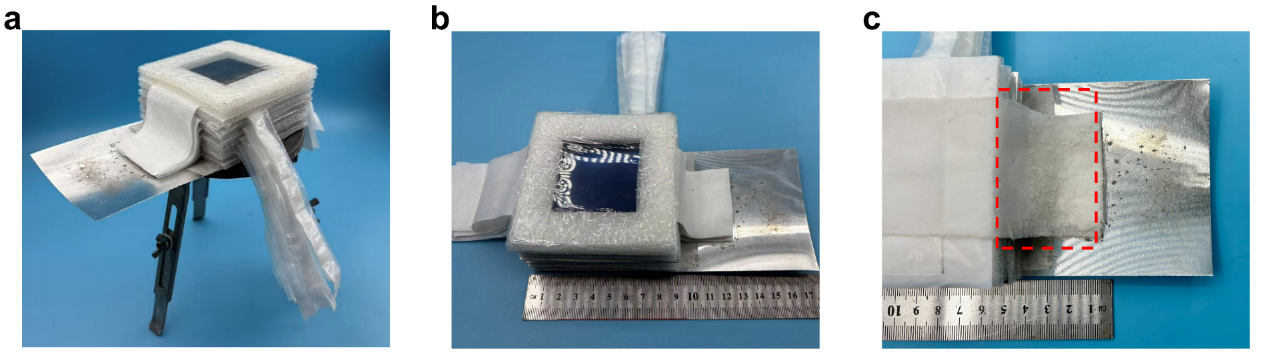


**Fig. S25** The passive multistage solar distiller for saline soil remediation. **a, b** Photograph of the multistage solar distiller. **c** Photograph of the salt crystallization zone of the device after 8 hours of operation on saline soil. The red dashed line indicates the location of salt crystallization.


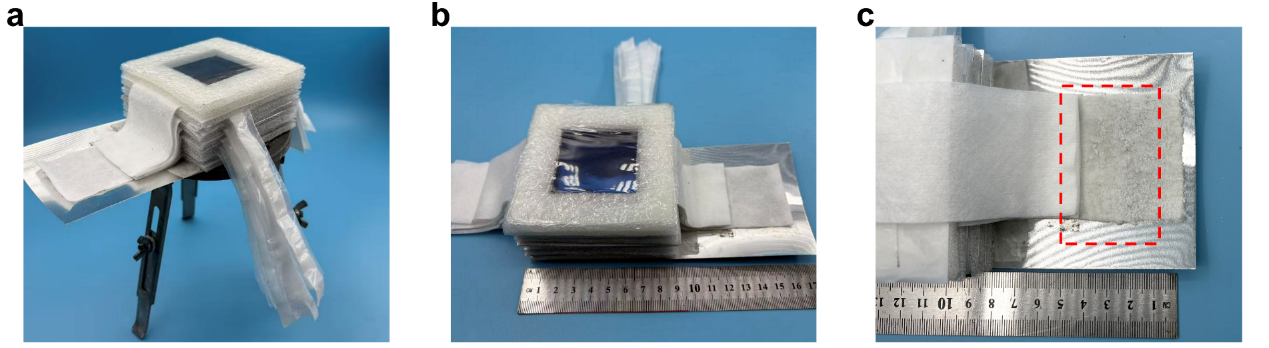


**Fig. S26** A replaceable fabric was incorporated beneath the extended zone of the solar distiller to serve as the salt collection layer. **a, b** Photograph of the multistage solar distiller with a salt collection layer. **c** Photograph of salt crystallization on the salt collection layer after 8 hours of operation on saline soil. The red dashed line indicates the location of salt crystallization.


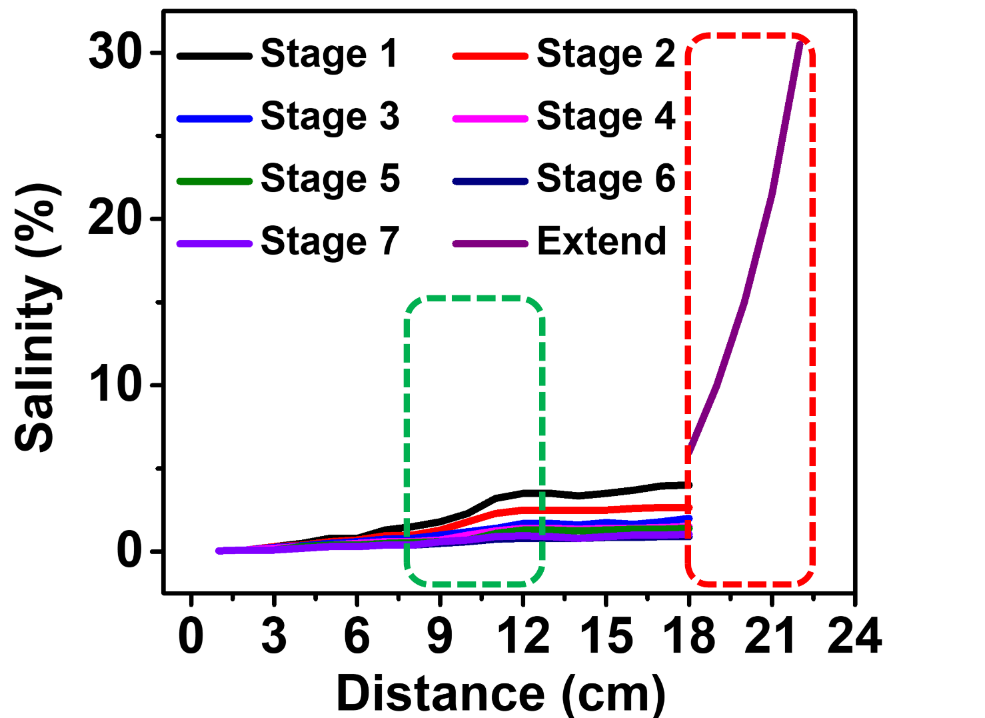


**Fig. S27** Salinity distribution in multistage solar distiller. The green dashed line indicates the salinity within the evaporation zone and the red dashed line indicates the salinity of the replaceable fabric.


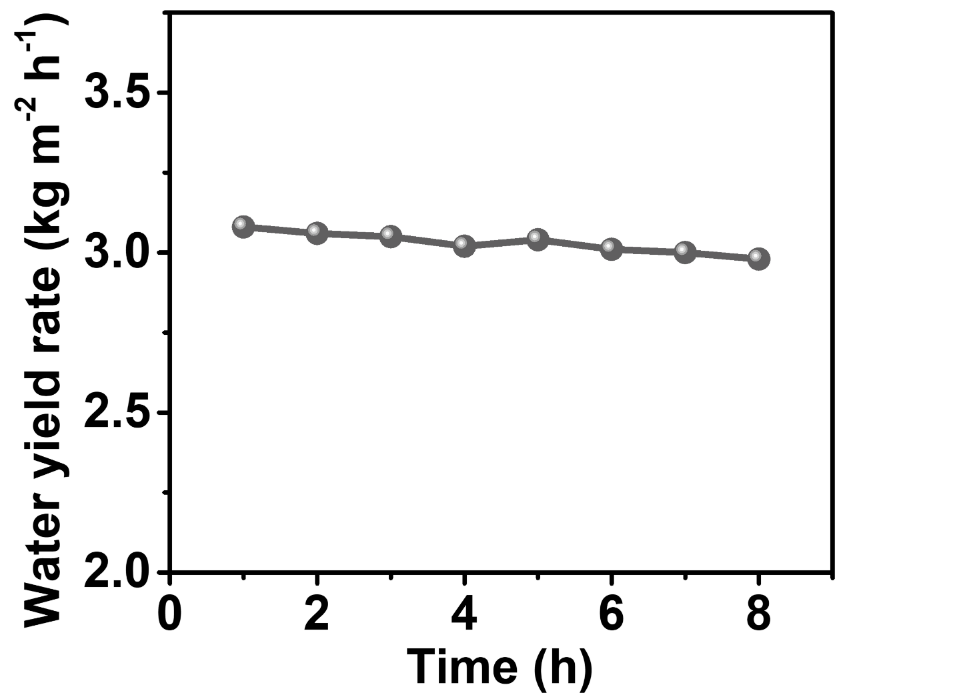


**Fig. S28** Time-dependent water yield of the multistage solar distiller during laboratory-scale soil desalination. The water yield of the multistage solar distiller remains stable at around 3 kg m^-2^ h^-1^ over 8 consecutive hours.


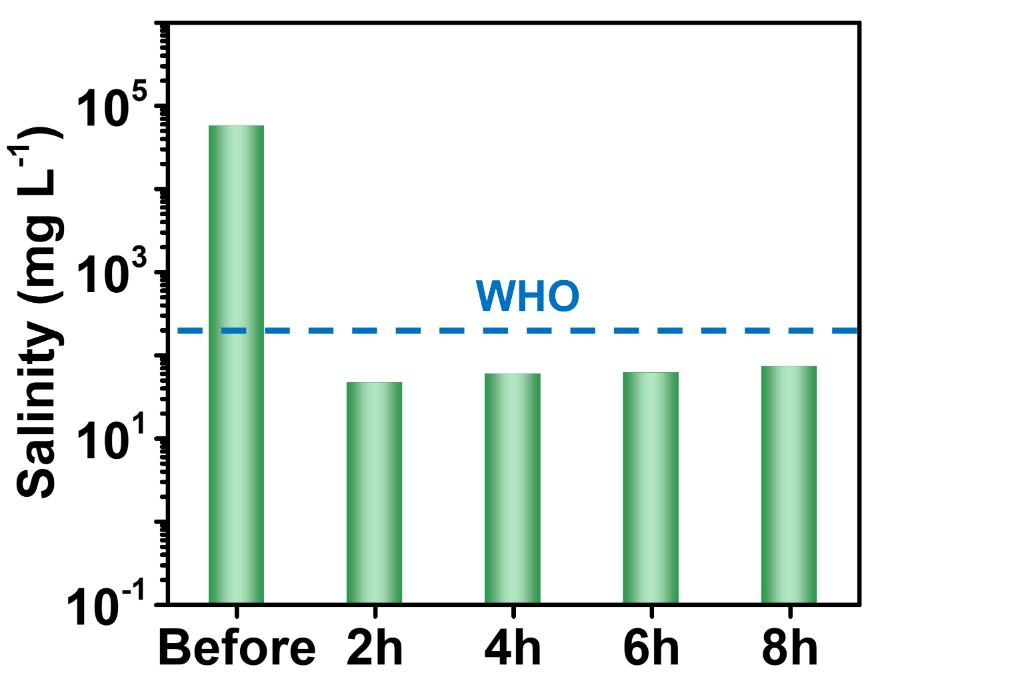


**Fig. S29** Water salinity (conductivity) of the produced water from the multistage solar distiller during laboratory-scale soil desalination.


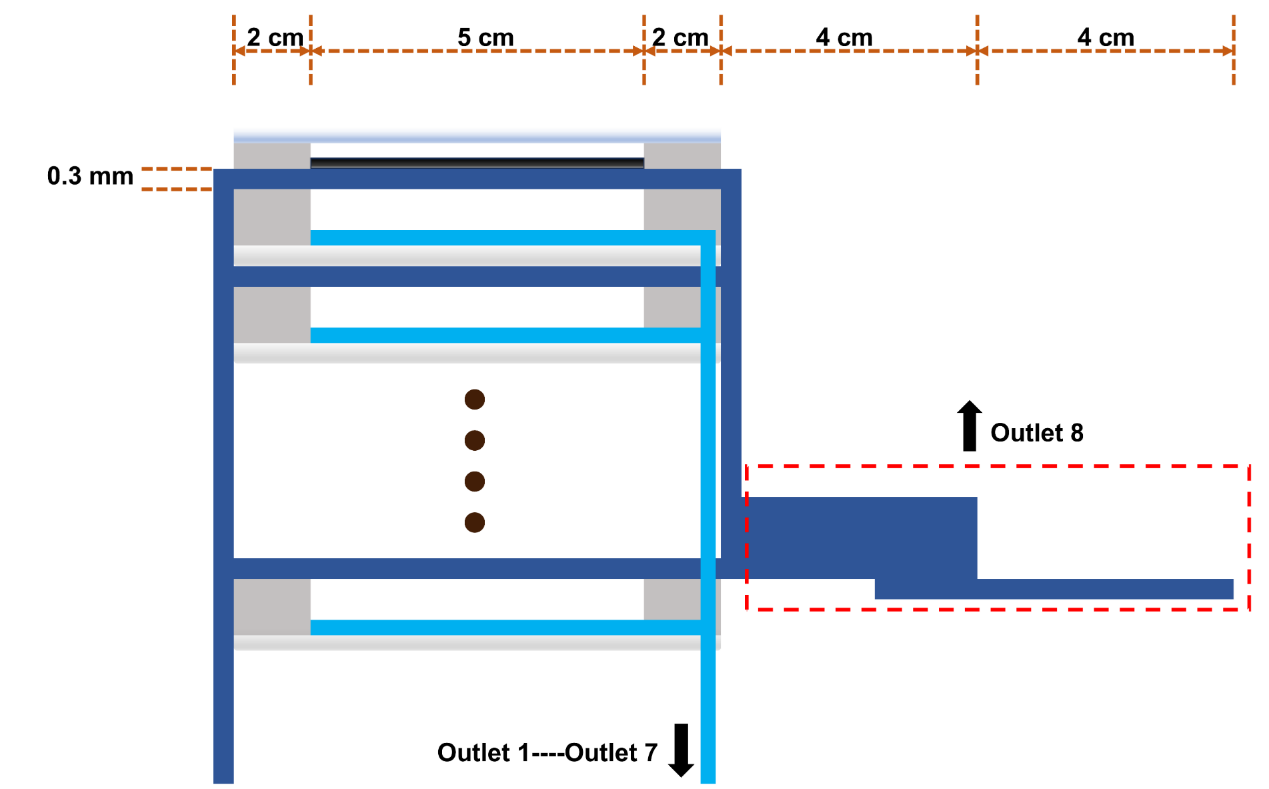


**Fig. S30** Model for COMSOL simulation of the multistage solar distiller.


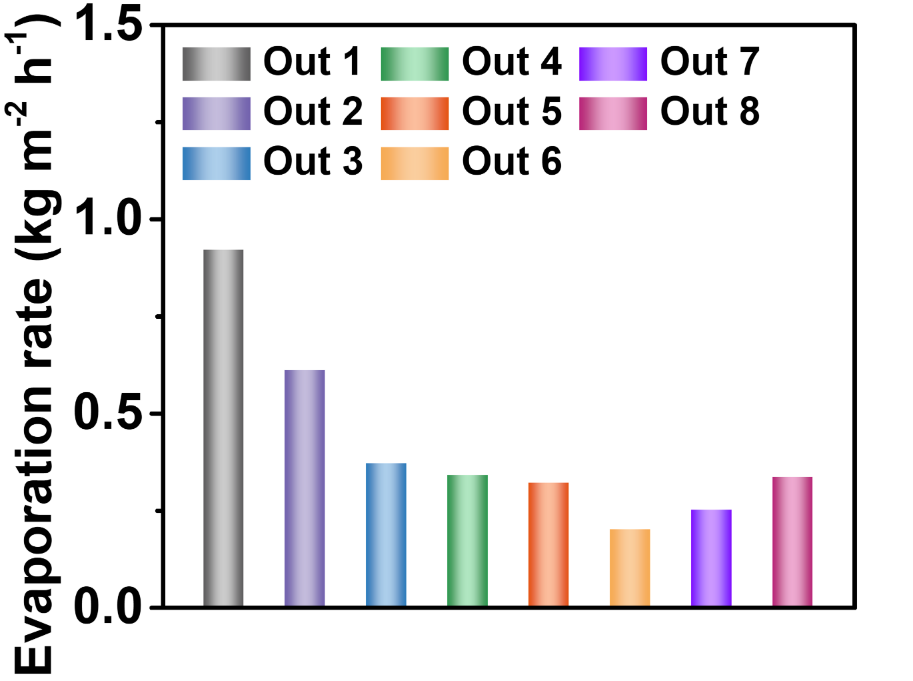


**Fig. S31** Evaporation rates of the outlet zone of the multistage solar distiller, used as input parameters for COMSOL simulations.


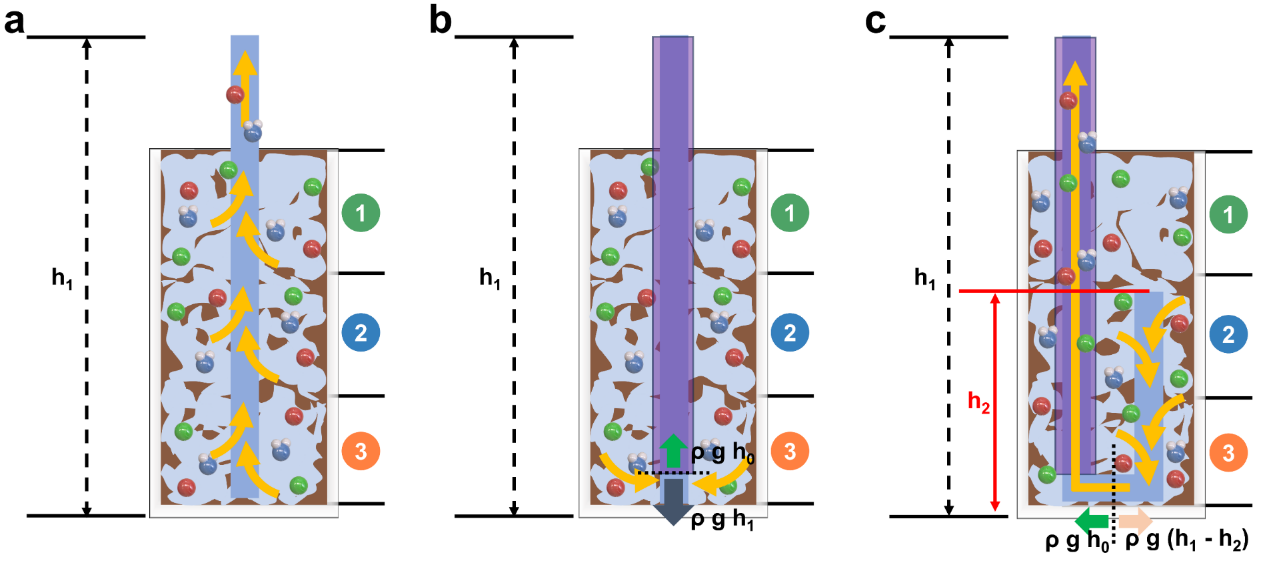


**Fig. S32** Schematic diagram of the U-shaped design for deep soil desalination. The fabric without any treatment **a**, the fabric with sealed upper part and exposed lower end **b** and the U-shaped fabric with sealed on the left and exposed on the right **c** were buried into the 30 cm-thick saline soil. h_0_ denotes the limiting height of water absorption of the fabric. h_1_ represents the water lifting height required to achieve soil desalination at a depth of 30 cm. h_2_ denotes the height of the right-hand side of the U-shaped fabric.


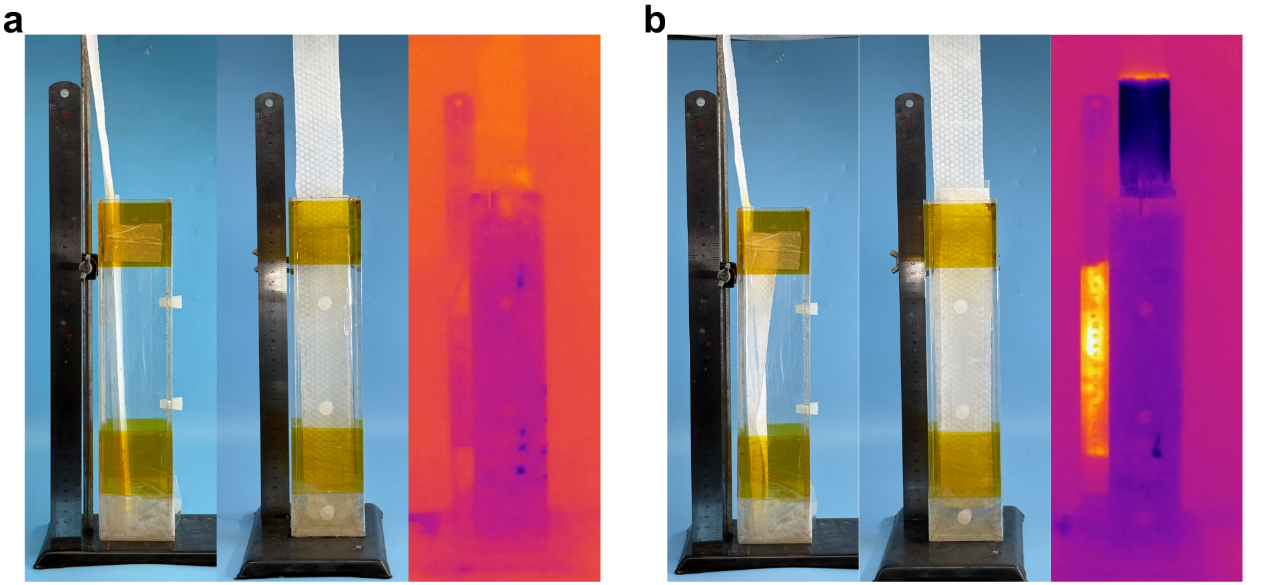


**Fig. 33** Photographs and thermal infrared images of soil water lifting by the fabric with sealed upper part and exposed lower end **a** as well as the U-shaped fabric with sealed on the left and exposed on the right **b**. To demonstrate the morphology of the fabric beneath the soil, we replaced the soil with water.


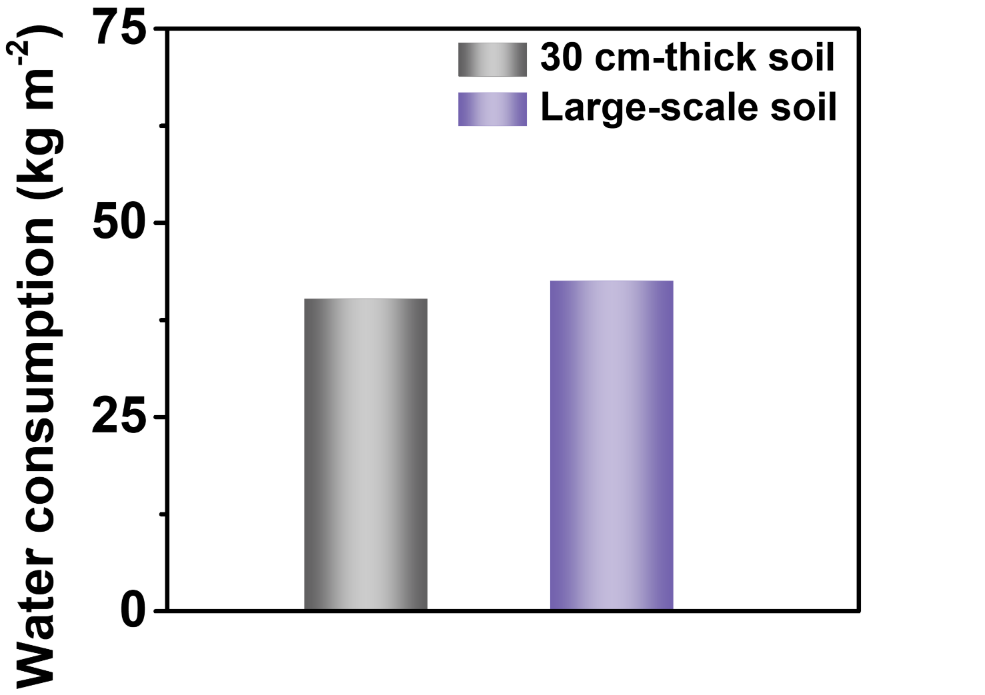


**Fig. S34** **a** Water consumption of the 5 cm × 5 cm multistage solar distiller during laboratory-scale soil desalination. **b** Water consumption of the 50 cm × 50 cm multi-stage solar distiller during outdoor large-scale soil desalination.


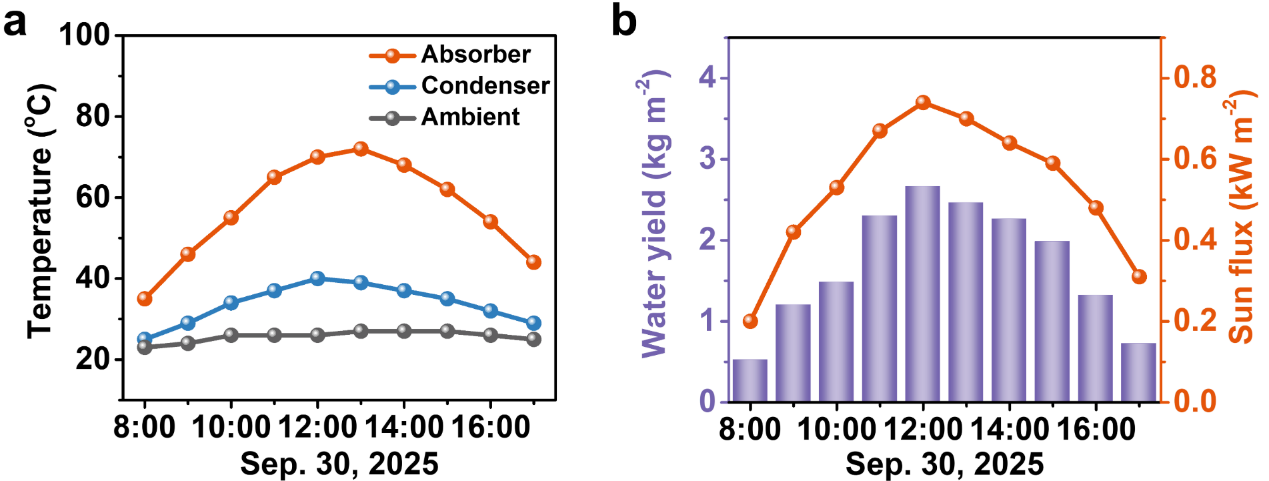


**Fig. S35** The 50 cm × 50 cm multistage solar distiller was first used to desalinate 0.6 M NaCl brine outdoors. **a** Temperature distribution. **b** Solar irradiance and hourly water production.

**Table S1** Simulation parameters of the COMSOL simulatio

| Parameter | Value |
| --- | --- |
| Water density  Water dynamic viscosity  Classical diffusion coefficient  Effective porosity | 997 kg m^-3^  8.9×10^-4^ Pa s  1.5×10^-9^ m^2^ s^-1^  90% |
| Ambient temperature  c_0_  c_1_ | 25°C  0.6 mol L^-1^  6 mol L^-1^ |

**Table S2** Simulation parameters of the COMSOL simulation for model-Ⅰ

| Parameter | Value |
| --- | --- |
| Velocity at the outlet 1 | 2.44×10^-7^ m s^-1^ |

**Table S3** Simulation parameters of the COMSOL simulation for model-Ⅱ

| Parameter | Value |
| --- | --- |
| Velocity at the outlet 1 | 2.57×10^-7^ m s^-1^ |

**Table S4** Simulation parameters of the COMSOL simulation for model-Ⅲ

| Parameter | Value |
| --- | --- |
| Velocity at the outlet 1  Velocity at the outlet 2 | 2.47×10^-7^ m s^-1^  0.93×10^-7^ m s^-1^ |

**Table S5** Simulation parameters of the COMSOL simulation for multistage solar distiller

| Parameter | Value |
| --- | --- |
| Velocity at the outlet 1  Velocity at the outlet 2  Velocity at the outlet 3  Velocity at the outlet 4  Velocity at the outlet 5  Velocity at the outlet 6  Velocity at the outlet 7  Velocity at the outlet 8 | 2.50×10^-7^ m s^-1^  1.75×10^-7^ m s^-1^  1.06×10^-7^ m s^-1^  0.92×10^-7^ m s^-1^  0.86×10^-7^ m s^-1^  0.53×10^-7^ m s^-1^  0.69×10^-7^ m s^-1^  0.93×10^-7^ m s^-1^ |

**Table S6** Simulation parameters for multistage solar distiller

| Parameter | Value |
| --- | --- |
| q_sun_  h_fg_  D_a_  ε  h_a_  k_a_  k_side_  k_water_  T_∞_  T_b_  t  a  b  h  C_water_ | 1000 W m^-2^  2350 kJ kg^-1^  3.0×10^-5^ m^2^ s^-1^  0.05  5.6 W m^-2^ K^-1^  0.027 W m^-1^ K^-1^  0.05 W m^-1^ K^-1^  0.6 W m^-1^ K^-1^  30℃  30℃  20 mm  50 mm  5 mm  0.3 mm  4.2 kJ kg^-1^ K^-1^ |

**Table S7** Saline soil classification standard [S1, S2]

| Soil salinity | Saline soil level | Suitable for cultivation |
| --- | --- | --- |
| 1.0-2.0 g kg^-1^ | Lightly saline soil | Hordeum wulgare etc. |
| 2.0-4.0 g kg^-1^ | Moderately saline soil | Vicia faba etc. |
| 4.0-6.0 g kg^-1^  >6.0 g kg^-1^ | Heavily saline soil  Saline soil | Lolium perenne etc.  None |

**Table S8** Comparison of experimental parameters between the multistage solar distiller of this study and interfacial evaporation system

| Parameter category | This study | Interfacial Evaporation |
| --- | --- | --- |
| Evaporator structure  Evaporation area | **7-stage solar still**  5 cm × 5 cm | Interfacial evaporator  4 cm × 4 cm |
| Soil volume  Soil Type | 6 cm × 6 cm × 5 cm  Coastal saline soil | 5 cm × 5 cm × 5 cm  Coastal saline soil |
| Irradiance  Ambient temperature  Relative humidity  Evaporation rate  Operating duration  Water consumption  Desalination rate | 1 sun (1 kW m^-2^)  25°C  50% RH  3.0 kg m^-2^ h^-1^  6 days  16.7 kg m^-2^  93.4% | 1 sun (1 kW m^-2^)  25°C  50% RH  1.34 kg m^-2^ h^-1^  10 days  36 kg m^-2^  94.8% |

**Table S9** Comparison of Cost and Energy consumption

| Method | Device cost  ($) | Energy consumption (kWh) | Operation cost ($) |
| --- | --- | --- | --- |
| Irrigation leaching  Interfacial evaporator  Multistage solar still | 359.60  592.84  368.65 | 19.38  0  0 | 12.90  2.63  1.22 |

**Supplementary References**

1. Market Supervision and Administration Bureau of Zhejiang Province. Technical regulations for afforestation of muddy saline land in coastal area: DB33/T1318(2023).
2. Market Supervision and Administration Bureau of Jiangsu Province. Technical code of practice for biological improvement for salinized soil: DB32/T5297(2025).
